# Anti-bacterial Effects of MnO_2_ on the Enrichment of Manganese-oxidizing Bacteria in Downflow Hanging Sponge Reactors

**DOI:** 10.1264/jsme2.ME20052

**Published:** 2020-09-19

**Authors:** Shuji Matsushita, Takafumi Hiroe, Hiromi Kambara, Ahmad Shoiful, Yoshiteru Aoi, Tomonori Kindaichi, Noriatsu Ozaki, Hiroyuki Imachi, Akiyoshi Ohashi

**Affiliations:** 1 Department of Civil and Environmental Engineering, Graduate School of Advanced Science and Engineering, Hiroshima University, 1–4–1, Kagamiyama, Higashi-Hiroshima, Hiroshima 739–8527, Japan; 2 Western Region Industrial Research Center, Hiroshima Prefectural Technology Research Institute, 2–10–1, Aga-minami, Kure, Hiroshima 737–0004, Japan; 3 Center of Technology for the Environment, Agency for the Assessment and Application of Technology, Geostech Building, Kawasan PUSPIPTEK, Serpong, Tangerang Selatan 15314, Indonesia; 4 Environmental Microbiology Laboratory, Graduate School of Advance Sciences of Matter, Hiroshima University, 2–313, Kagamiyama, Higashi-Hiroshima, Hiroshima 739–8527, Japan; 5 Department of Subsurface Geobiological Analysis and Research, Japan Agency for Marine-Earth Science & Technology, Yokosuka, Kanagawa 237–0061, Japan

**Keywords:** anti-bacterial effects, biological manganese oxidation, biological manganese oxide, downflow hanging sponge reactor, metal oxide

## Abstract

We focused on the use of abiotic MnO_2_ to develop reactors for enriching manganese-oxidizing bacteria (MnOB), which may then be used to treat harmful heavy metal-containing wastewater and in the recovery of useful minor metals. Downflow hanging sponge (DHS) reactors were used under aerobic and open conditions to investigate the potential for MnOB enrichment. The results of an experiment that required a continuous supply of organic feed solution containing Mn(II) demonstrated that MnOB enrichment and Mn(II) removal were unsuccessful in the DHS reactor when plain sponge cubes were used. However, MnOB enrichment was successful within a very short operational period when sponge cubes initially containing abiotic MnO_2_ were installed. The results of a microbial community analysis and MnOB isolation revealed that MnOB belonging to *Comamonadaceae* or *Pseudomonas* played a major role in Mn(II) oxidation. Successful MnOB enrichment was attributed to several unidentified species of *Chitinophagaceae* and *Gemmataceae*, which were estimated to be intolerant of MnO_2_, being unable to grow on sponge cubes containing MnO_2_. The present results show that MnO_2_ exerted anti-bacterial effects and inhibited the growth of certain non-MnOB groups that were intolerant of MnO_2_, thereby enabling enriched MnOB to competitively consume more substrate than MnO_2_-intolerant bacteria.

Manganese is a transition metal that is present in large quantities throughout the earth. The majority of manganese exists in the form of manganese oxides, such as MnO_x_. MnO_2_ has a high adsorption capacity for many cationic metals and is closely involved in the global cycling of various elements (Tebo *et al.*, 2005). The formation of Mn oxide in biological reactions progresses at several orders of magnitude greater than that of chemical reactions at approximately neutral pH values in general biological wastewater treatment environments (Bargar *et al.*, 2000; Morgan, 2005). Therefore, Mn oxidation in various environments is considered to be associated with the presence of microbes (Tebo *et al.*, 1984; Tipping, 1984). A phylogenetically diverse group of bacteria (Tebo *et al.*, 2004; Barboza *et al.*, 2015) and fungi (Tani *et al.*, 2004a) perform Mn oxidation. By using these microorganisms to produce biogenic MnO_x_ (Bio-MnO_x_), it may be possible to remove harmful metals, such as As(V) and Pb(II), from contaminated water (Tani *et al.*, 2004b; Miyata *et al.*, 2007) while recovering useful metals, such as Co(II), Zn(II), and Ni(II) (Tani *et al.*, 2004a).

Despite the detection of several heterotrophic bacteria exhibiting Mn(II)-oxidizing properties in various environments, difficulties are associated with preferentially cultivating manganese-oxidizing bacteria (MnOB) in reactors that are open to the atmosphere. A previous attempt to continuously cultivate MnOB in a downflow hanging sponge (DHS) reactor, to which a synthetic organic substrate was supplied, was unsuccessful (Cao *et al.*, 2015); MnOB reportedly failed to effectively compete with other bacteria for the substrate under eutrophication conditions because the majority of MnOB had been detected in oligotrophic environments. Therefore, we attempted to develop a unique method to enrich MnOB coupled to nitrifiers by naturally and slowly providing small quantities of soluble microbial products (SMPs) accompanied by the nitrification process. As a result, MnOB enrichment was successful; however, a very long operation period was necessary. Using the same methodology, Matsushita *et al.* (2018) also enriched MnOB in a methane oxidation reactor. They observed the suppression of methane oxidation activity by Bio-MnO_x_ that accumulated on sponge carriers in the reactor. They also found that abiotic MnO_2_ inhibited some methane-oxidizing bacteria in a batch test.

MnOB need to have a high tolerance for abiotic MnO_2_ because they produce Bio-MnO_x_ and tolerate its inhibitory effects. MnO_2 _may also inhibit the activities of other non-MnOB, such as *Escherichia coli* (Sawai, 1985). We previously found that upon using a medium containing peptone and yeast extract, the consumption rate of activated sludge decreased in the presence of abiotic MnO_2_ in a preliminary batch experiment (data not shown). Moreover, we measured the colony-forming units of activated sludge in a plate culture with abiotic MnO_2_ and found 99.6% less growth than that in the same plate culture without abiotic MnO_2_. In our preliminary experiment, 10.7% of MnO_2_-tolerant colonies were estimated to be MnOB (Table S1). Based on these findings, we hypothesized that if abiotic MnO_2_ is initially added to reactors, MnOB enrichment will occur within a short operational period. Hence, we attempted to demonstrate that DHS reactors with abiotic MnO_2_-installed sponges will induce MnOB enrichment using three different organic substrates (Shoiful *et al.*, 2020). However, we did not conduct a negative control experiment using a DHS reactor with plain sponges, and, thus, no comparisons of Mn(II) oxidation performance and microbial communities were conducted between plain and abiotic MnO_2_-installed sponges. Therefore, the extent to which installed MnO_2_ influences MnOB enrichment at the start as well as the bacteria whose growth is inhibited currently remain unclear.

We herein confirmed early MnOB enrichment using an organic feed medium in two DHS reactors, in which sponge carriers containing abiotic MnO_2 _were installed. MnOB successfully competed with non-MnOB for the substrate and Mn(II) oxidation was achieved within a short period during the start-up phase. We attempted to identify the bacteria whose growth was suppressed by the inhibitory effects of MnO_2_ after comparisons of the growth levels of the microbial community in another DHS reactor in which abiotic MnO_2 _was not installed. Several MnOB isolated from the reactor have also been discussed, along with the predominant MnOB involved in Mn(II) oxidation.

## Materials and Methods

### Experimental set-up

Three DHS reactors that were identical, except for the installation of abiotic MnO_2_, were used in the present study (Fig. S1). Two L columns (height, 100‍ ‍cm; inner diameter, 5‍ ‍cm) and a string of 20 polyurethane sponge cubes (each volume, 2×2×2‍ ‍cm^3^; total volume, 160‍ ‍cm^3^; pore volume ratio, 96%) were diagonally connected to each other in a series. Activated sludge collected from an aeration tank in a municipal wastewater treatment plant was used as the inoculum. Before setting up the DHS reactors, the sponges in Run 1 were soaked in diluted activated sludge. In Runs 2 and 3, sponges squeezed and soaked in a suspension liquid mixed with activated sludge and abiotic MnO_2 _(first grade, powder; Kishida Chemical) were used for inoculation and MnO_2 _installation. Based on the sponge volume, the abiotic MnO_2_ concentration selected was 100‍ ‍g L^–1^ using inhibition data in the preliminary batch experiment (data not shown). Prior to the application of abiotic MnO_2_, a Mn(II) adsorption pre-treatment was conducted in a large amount of solution with 5‍ ‍mg Mn(II) L^–1^ to prevent Mn(II) adsorption on MnO_2_ in the reactors. Before operating the reactor, 5‍ ‍mg Mn(II) L^–1^ of the feed solution without organic substances was supplied to the reactor at high and low water flow rates; we confirmed that no additional Mn(II) adsorption occurred in the reactor because its concentrations in the influent and effluent were the same.

### Substrate composition

Chemical oxygen demand (COD) was used as an indicator of the amount of organic matter. Substrates with concentrations ranging from 50–100‍ ‍mg COD L^–1^ were prepared at a 4:1 ratio (w/w) of polypeptone and yeast extract prepared using tap water. Mn(II) (MnCl_2_·4H_2_O) was added to substrates in the range of 0‍–‍5‍ ‍mg‍ ‍Mn‍ ‍L^–1^, along with minerals including FeSO_4_·7H_2_O (0.1‍ ‍mg‍ ‍L^–1^), K_2_HPO_4 _(29.9‍ ‍mg L^–1^), Na_2_HPO_4_·12H_2_O (223.23‍ ‍mg‍ ‍L^–1^), CaCl_2_·2H_2_O (0.05‍ ‍mg L^–1^), MgSO_4_·7H_2_O (0.2‍ ‍mg‍ ‍L^–1^), CuSO_4_·5H_2_O (0.0025‍ ‍mg L^–1^), Na_2_SeO_4_ (0.005‍ ‍mg‍ ‍L^–1^), NiCl_2_·6H_2_O (0.019‍ ‍mg L^–1^), CoCl_2_·6H_2_O (0.024‍ ‍mg L^–1^), Na_2_MoO_4_·2H_2_O (0.022‍ ‍mg L^–1^), H_3_BO_3_ (0.001‍ ‍mg‍ ‍L^–1^), and ZnSO_4_ (0.043‍ ‍mg L^–1^). pH was adjusted to 7.8 using 0.02‍ ‍mM phosphate buffer. We also confirmed that chemical Mn(II) oxidation did not occur in the DHS reactor in the absence of inoculated abiotic MnO_2_ in these substrates.

### Operating conditions

All reactors were operated in a temperature-controlled dark room at 25°C (Table 1). The substrate was fed into the top of the reactor at a hydraulic retention time (HRT) of 0.25–4.25 h based on the sponge volume. The effluent was recirculated at a ratio of 9:1 with the influent. Air was supplied from the upper end of each reactor at a flow rate of 0.5 L min^–1^ to maintain an aerobic environment. The difference between Runs 2 and 3 was the manner in which Mn(II) was supplied. In Runs 2 and 3, Mn(II) was provided from the start-up phase and from day 67 onwards, respectively.

### Analytical methods

Wastewater influents and effluents were routinely sampled twice every week for 127 days. Water samples were filtered using PTFE membranes with a pore size of 0.2‍ ‍μm, after which Mn(II) concentrations in the influent and effluent were measured using the periodate oxidation method with a Hach water quality analyzer (DR-2800; Hach). The particulates produced in the reactor were identified as Mn oxides using Leucoberbeline Blue I (LBB; Sigma-Aldrich) (Krumbein and Altmann, 1973; Zhu *et al.*, 2017).

### Microbial community analysis

To perform a microbial community analysis, biomass sampling was conducted by squeezing the sponge carriers of the upper, middle, and lower parts used during all Runs on day 35 and squeezing those on day 73 for Run 3. The collected biomass was washed with phosphate buffer. DNA was extracted using the FastDNA^®^ SPIN Kit for Soil (MP Biomedicals) and its concentration was adjusted to 2‍ ‍ng μL^–1^. PCR amplification of the 16S rRNA gene was performed using the primer set containing 341F (5′-CCTACGGGNGGCWGCAG-3′) and 805R (5′-GACTACHVGGGTATCTAATCC-3′), with KAPA Hifi DNA polymerase (Nippon Genetics). PCR was performed by initial denaturation at 95°C for 3‍ ‍min, followed by 35 cycles at 95°C for 30‍ ‍s, at 55°C for 30‍ ‍s, and at 72°C for 30‍ ‍s; the final extension was performed at 72°C for 5‍ ‍min. PCR products were purified and sequenced by the emulsion method using Illumina/Miseq (Illumina) at Hokkaido System Science. The pair-end sequences obtained were analyzed using QIIME (v1.8.0) (Caporaso *et al.*, 2010). Operational taxonomic units (OTUs) were grouped based on a threshold value of 97% identity for DNA using the UCLUST method (Edgar, 2010); these OTUs were classified using the Greengenes database (McDonald *et al.*, 2012; Werner *et al.*, 2012).

### Isolation and identification of MnOB

To identify the bacteria involved in Mn(II) oxidation, the isolation of MnOB was attempted using a biomass sample that was collected by squeezing sponges removed from the middle section of the reactor during Run 2 on day 198. Run 2 was continued and an HRT of 4.25 h was maintained until day 198 for performing the isolation process. The sampled biomass was inoculated on solid agar plates (15‍ ‍g L^–1^) containing medium (peptone 0.2‍ ‍g L^–1^, yeast extract 0.05‍ ‍g L^–1^, and HEPES 10‍ ‍mM; pH 7.2) and MnCl_2_·4H_2_O (55‍ ‍mg Mn[II] L^–1^) using a platinum loop and cultured at 25°C. After 1 or 2 days, approximately 50 of the colonies that formed on each plate were picked using a toothpick, and each colony was inoculated into a liquid medium (peptone 0.2‍ ‍g L^–1^, yeast extract 0.05‍ ‍g L^–1^, and HEPES 10‍ ‍mM; pH 7.2). The liquid medium was agitated at 100 rpm and 25°C for 1 day. Cultivated bacteria were inoculated on an identical plate containing a solid medium to isolate pure bacteria. To confirm whether the isolates exhibited Mn(II)-oxidizing activity, we conducted LBB spot tests (López-Cortés and del Pliego, 1998) using isolated colonies once every week for 3‍ ‍weeks. This evaluation method was repeated five times. After a blue color was observed, the isolate was identified as MnOB.

### Identification of isolated bacteria

The DNA sequences of the 16S rRNA genes for every isolated bacterial strain were elucidated. PCR amplification was performed using 25‍ ‍μL 1×KOD buffer, 2‍ ‍mM dNTPs, 0.3‍ ‍μM primers, and 0.1-unit KOD FX (Toyobo). The 27F (5′-AGAGTTTGATCCTGGCTCAG-3′) and 1492R (5′-GGTTACCTTGTTACGACTT-3′) primer set was used (De Long, 1992). PCR was performed by conducting the initial denaturation step at 95°C for 3‍ ‍min, followed by 35 cycles at 95°C for 30‍ ‍s, at 55°C for 30‍ ‍s, and at 72°C for 30 s; a final extension step was performed at 72°C for 5‍ ‍min. PCR products were purified using the FastGene Gel/PCR Extraction Kit (Nippon Genetics) and subjected to direct sequencing by the Sanger method using 518F (5′-CCAGCAGCCGCGGTAATACG-3′) and/or 800R (5′-TACCAGGGTATCTAATCC-3′) primers (Jacob and Irshaid, 2012). Primer sequences and low-quality regions were removed from each obtained sequence using Chromas gene analysis software (v2.6; Technelysium Pty). Contigs were prepared using the genetic analysis software Genestudio ™ Professional Edition (v2.2.0.0; GeneStudio). The sequence obtained of at least 600 bp or more was confirmed to exhibit homology of more than 98% via BLASTN (Johnson *et al.*, 2008). The 16S rRNA gene DNA sequences of each isolated strain were deposited in GenBank (accession numbers LC273429–LC273512).

## Results

### Mn(II) oxidation and MnOB enrichment

**Fig. 1.**
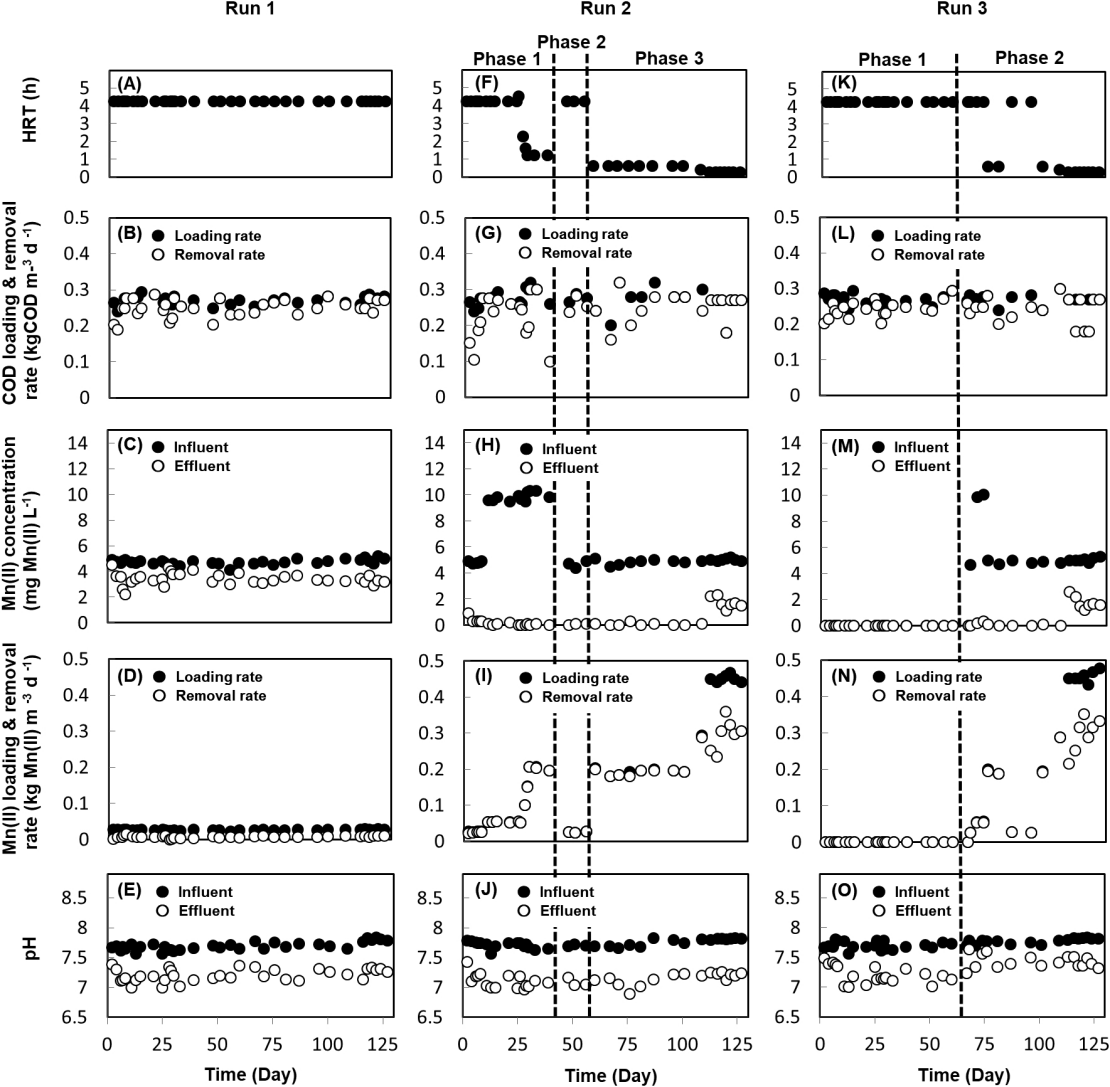
Time-course analysis of DHS reactor performance in Runs 1, 2, and 3. (A), (F), and (K): HRT. (B), (G), and (L): COD loading and removal rates. (C), (H), and (M): Mn(II) influent and effluent concentrations. (D), (I), and (N): Mn(II) loading and removal rates. (E), (J), and (O): pH values.

The three DHS reactors (R1, R2, and R3) were constantly operated at the same COD loading rate of approximately 0.27 kg COD m^–3^ d^–1^ (based on sponge volumes) for 127 days; however, the concentrations of the organic substrate and HRT differed between the phases of the three runs (Runs 1, 2, and 3). High COD removal was observed from the start-up phase and an average of approximately 90% was noted for all Runs (Fig. 1B, G, and L).

In Run 1, which was performed in R1, no abiotic MnO_2_ was included in the Mn(II) concentration of 5‍ ‍mg L^–1^ for the influent, and the value was slightly reduced in the effluent; however, the Mn(II) removal rate was not enhanced, even after a long operational period of 127 days (Fig. 1C and D). The Mn(II) removal process needs to consist of three factors: Mn(II) oxidation, Mn(II) adsorption onto the biomass, and Mn(II) adsorption onto MnO_2_. This slight reduction in the Mn(II) concentration may be caused by Mn(II) adsorption on the formed biofilm; the color of the biofilm did not change to black and the deposition of MnO_2_ particles was not observed at the bottom of the DHS reactor, indicating that Bio-MnO_x _had not accumulated.

In contrast, MnOB enrichment and Mn(II) oxidation were successfully performed during Run 2 in R2, in which a sponge containing abiotic MnO_2_ was used. As the substrate was fed, Mn(II) removal occurred rapidly and nearly 100% removal was observed at the low Mn(II) loading rate of 0.03‍ ‍kg Mn(II) m^–3^ d^–1^ (Fig. 1H and I). We then increased the Mn(II) loading rate on day 8 by increasing the Mn(II) concentration of 5‍ ‍mg L^–1^ in the influent twice and decreasing the HRT from 4.25 h to 1 h. Nevertheless, nearly complete Mn(II) removal was performed. During these 30 days, from day 27 onwards, the Mn(II) loading rate increased stepwise to 0.20 kg Mn(II) m^–3^ d^–1^. Mn(II) was removed without a significant increase being detected in the Mn(II) concentration in the effluent. This relatively high performance suggested that a sufficient level of MnOB enrichment was achieved during the short one-month duration, which was similar to that previously achieved under similar conditions (Shoiful *et al.*, 2020).

In phase 2 of Run 2, after the Mn(II) loading rate was reduced to 0.03‍ ‍kg Mn(II) m^–3^ d^–1^ for 16 days, the loading rate was the same as that on day 56. Although the change in the Mn(II) loading rate was significant, the Mn(II) removal rate immediately recovered (Fig. 1H and I). This result suggests that MnOB activity was maintained in the absence of Mn(II). To investigate the maximum Mn(II) removal ability, we gradually increased the Mn(II) loading rate in phase 3 (day 57–127). At the maximum Mn(II) loading rate of 0.47‍ ‍kg Mn(II) L^–1^ d^–1^, a Mn(II) concentration of approximately 1.6‍ ‍mg L^–1^ was detected in the effluent, which corresponded to 87% removal; R2 had a Mn(II) removal capacity of 0.36 kg Mn(II) L^–1^ d^–1^ (Fig. 1H and I). Furthermore, deposited particles accumulated at the bottom of R2 (Photo S2), which were confirmed to be MnO_x_ compounds by LBB, suggesting that they were derived from newly formed Bio-MnO_x_ by Mn(II) oxidation.

Although R3 and R2 were identical, Run 3 performed in R3 differed from Run 2 in the manner of supplying Mn(II). In phase 1 of Run 3 (day 0–67), Mn(II) was not supplied because it was unclear whether MnOB had been successfully cultivated. Furthermore, MnO_x_ particles were not deposited at the bottom of R3 until day 67. As soon as Mn(II) was provided from day 68 during phase 2 and the Mn(II) loading rate had rapidly increased, the removal of influent Mn(II) was nearly 100% and the Mn(II) loading rate became 0.2 kg Mn (II) L^–1^ d^–1^ within only 8 days (Fig. 1M and N). This performance indicated that MnOB were successfully enriched in the absence of Mn(II) under abiotic MnO_2_ conditions. In addition, MnOB activity remained unaffected by the Mn(II) load change (day 81–96), which was similar to the result for Run 2. By imposing a high Mn(II) loading rate from day 109, the Mn(II) removal rate gradually increased to 0.35 kg Mn(II) L^–1^ d^–1^, which was achieved on day 122 (Fig. 1N); this rate was similar to that associated with the maximum capacity of R2. The results of Runs 1–3 demonstrated that MnOB enrichment was possible on organic substrates, including the continuously supplied medium containing peptone and yeast extract, and clearly indicated that MnO_x_ installation in the reactor was very effective for the enrichment process.

### Bacterial community analysis

The microbial communities involved in biomass samples for Run 1 (SR1), Run 2 (SR2), and Runs 3 (SR3.1 and SR3.2) were expected to significantly differ because MnOB were not anticipated to be present in biomass SR1. However, the compositions of the communities were very similar at the phylum level (Fig. S2). *Bacteroidetes* and *Proteobacteria* were dominant in all samples. A minor difference was that lower levels of *Armatimonadetes*, *Chlorobi*, and OD1 were detected in SR1 than in other Runs. This result indicates that the composition of the microbial community was primarily governed by the organic substrate, regardless of the presence of MnO_2_.

A list of the putative MnOB detected, which are phylogenetically close to known MnOB, although not all have a Mn(II) oxidation function, is shown in Table S2. The sums of the read numbers for these two bacteria groups were 20.5, 12.9, and 10.6% of the total read numbers for SR2, SR3.1, and SR3.2, respectively. Several MnOB on the list were responsible for Mn(II) oxidation in the bioreactor. The two bacterial groups dominated at 11.6%, even in SR1, during which MnOB was not expected to be detected. This result suggests that even if a bacterium is phylogenetically close to MnOB at the genus level, it is difficult to identify it as MnOB. Additionally, in the absence of abiotic MnO_2_ (Run 1), the presence of specific bacteria that have no ability to perform Mn(II) oxidation and are closely related to MnOB may be more dominant than MnOB, likely due to their higher growth rate. In contrast, under the abiotic MnO_2_ condition (Runs 2 and 3), MnOB were enriched instead of the specific above-mentioned bacteria, and this may have been because MnO_2_ exerts negative effects on the overall growth of specific bacteria.

It is important to note that MnOB may not always oxidize Mn(II) depending on the environment. Even if this speculation is true, the results obtained show that MnOB enrichment failed in Run 1 because the environment was almost the same as that in Runs 2 and 3, during which Mn(II) oxidation was observed.

### Isolation and identification of MnOB

We isolated 84 bacterial strains from the sponge biomass on day 198 of Run 2 to identify the strains involved in Mn(II) oxidation in the DHS reactor. In total, 74% of the isolated strains were confirmed to belong to the family *Comamonadaceae* by a phylogenetic analysis (Table 2), while the family *Comamonadaceae* accounted for less than 10% of the microbial community, according to the results of an analysis performed using next-generation sequencing (Table S2). Furthermore, the Mn oxidation capacity of the isolated bacteria was examined using the LBB spot test. Forty-eight strains were confirmed to be MnOB because they positively reacted with the formed MnO_x_, as represented by a color change to blue for each colony after three weeks. Among the isolated MnOB, three strains exhibited a high Mn(II) oxidation capacity because the deposition of brown Bio-MnO_x _was observed around the colonies within one week of cultivation (Table 2). These three strains, which belonged to the genus *Pseudomonas*, were classified into the *P. putida* group according to the classification system described in a previous study (Anzai *et al.*, 2000) (Fig. S3). *P. putida* is a well-studied MnOB and contains two multicopper oxidase (MCO) genes and an animal heme peroxidase gene involved in Mn(II) oxidation (Geszvain *et al.*, 2016), which may explain why isolated MnOB belonging to the *P. putida* group exhibited a high capacity for performing Mn oxidation.

Although the other isolates of the 45 strains were also estimated to be MnOB, their Mn(II)-oxidizing ability was very weak because a long cultivation time was required to confirm Bio-MnO_x_ production. A large proportion of these strains belonged to the genus *Comamonas* of *Comamonadaceae*. Among the 45 isolates with a weak Mn(II)-oxidizing capacity, three were classified into the genera *Mitsuaria*, *Ensifer*, and *Sphingomonas* (Table 2). One genus, *Delftia* belonging to *Comamonadaceae* and one genus, *Mitsuaria* are reportedly not able to oxidize Mn(II), which suggests that many undiscovered MnOB exist in nature. Hence, Mn(II) oxidation in the bioreactor may be performed by a wide range of MnOB with very different activities.

## Discussion

**Fig. 2.**
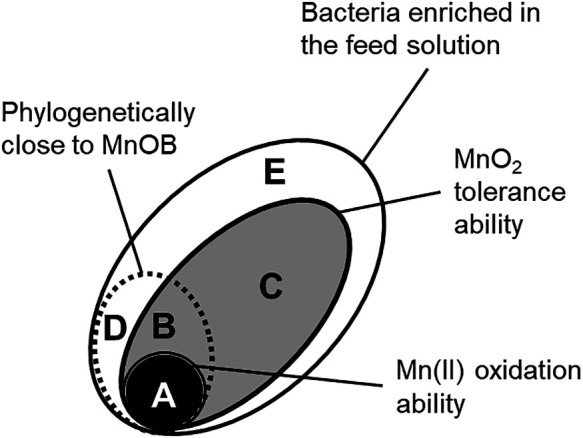
Classification of microbial communities with the ability to oxidize Mn(II) into five groups. A: MnOB, B: non-MnOB that exhibit MnO_2_ tolerance and are phylogenetically close to MnOB, C: non-MnOB that exhibit MnO_2_ tolerance and are not phylogenetically close to MnOB, D: strains lacking MnO_2_ tolerance that are phylogenetically close to MnOB, E: strains lacking MnO_2_ tolerance that are not phylogenetically close to MnOB.

We hypothesized that abiotic MnO_2_ inhibits the growth of bacterial groups competing with MnOB for organic substrates. We also postulated that early MnOB enrichment is possible in MnO_2_-installed reactors. As expected, the effects of MnO_2_ on MnOB enrichment were confirmed in the present study. All bacteria enriched in Runs 2 and 3 were expected to exhibit tolerance toward MnO_2_, while MnO_2_-tolerant bacteria and intolerant bacteria may both have existed in Run 1. In addition, all bacteria enriched in Run 1 were expected to lack the ability to oxidize Mn(II) because Mn(II) removal was markedly less than that in Runs 2 and 3. Therefore, the bacteria that grew on the substrate in the present study were classified into five (A–E) groups; A, MnOB; B, non-MnOB that exhibit MnO_2_ tolerance and are phylogenetically close to MnOB; C, non-MnOB that exhibit MnO_2_ tolerance and are not phylogenetically close to MnOB; D, strains lacking MnO_2_ tolerance that are phylogenetically close to MnOB; E, strains lacking MnO_2_ tolerance that are not phylogenetically close to MnOB (Fig. 2). It is possible to roughly estimate the groups that each of the bacteria belong to by comparing microbial communities between Runs 1, 2, and 3 (Table S3). For example, the bacteria detected in all runs that were tolerant to MnO_2_, but were not MnOB, belonged to group B or C. The bacteria present in Run 1, but absent in Run 2 or 3 may be affiliated with group D or E and be intolerant to MnO_2_. The bacteria present in Run 2 or 3, but absent in Run 1 may be tolerant to MnO_2_ and belong to group A, B, or C.

Of the 5,518 OTUs obtained from 300,837 sequence reads, a large number consisted of a few reads of strains that were supposedly uncultivable, difficult to cultivate directly on the substrate, or SMPs excreted by other bacteria. Therefore, 374 OTUs were ultimately used for classification after the elimination of the minor populations with read number ratios of less than 0.1% for each community, resulting in the population sizes shown in Table 3 for the five groups. The selected OTUs dominated 86.8% of all reads in an average of four samples.

Notably, in Run 1, groups B and C, which exhibited MnO_2_ tolerance, were the dominant groups, accounting for 10.7 and 65.7%, respectively, whereas MnO_2_ was neither initially provided nor produced (Table 3). Group C dominated all runs, accounting for more than 50%. These results suggest that even though bacteria exhibited MnO_2_ tolerance, not all bacteria were able to oxidize Mn(II) and may naturally occur in large quantities and exhibit diversity. The value for group E, which exhibited MnO_2_ intolerance, was 22.8%. The process of classification clearly shows that MnO_2_ inhibited the growth of MnO_2_-intolerant bacteria and that several unidentified species in the families *Chitinophagaceae* and *Gemmataceae*, which were predominant in the substrate, were presumably intolerant of MnO_2_ (Table S3). Furthermore, group D only accounted for 0.9%, which suggests that most of the bacteria were phylogenetically close to MnOB and tolerant of MnO_2_.

By suppressing only 23.7% of group D and E strains, the enrichment of MnOB from group A was successful. However, it was difficult to identify enriched MnOB by comparing the microbial communities employed in this analysis. *Comamonadaceae* and *Pseudomonadaceae* were enhanced in Runs 2 and 3 and are predicted to play a major role in Mn(II) oxidation (Table S3). Not all species belonging to *Comamonadaceae* or *Pseudomonadaceae* were MnOB because both were also detected, even in Run 1. Although the percentage of group A bacteria was less than 4.8% in Runs 2 and 3.2, a high Mn(II) removal rate of approximately 0.35 kg Mn(II) m^–3^ d^–1^ was achieved. If the operational conditions needed to eliminate groups B and C are identified, higher Mn(II) oxidation rates will be achieved. Moreover, we showed that some OTUs affiliated with *Planctomycetes* or *Armatimonadetes* were only detected when Mn(II) oxidation occurred during Runs 2 and 3.2 (Table S3), which suggests that these OTUs are unknown MnOB. This result also indicates that bacteria enhanced in Mn(II)-oxidizing environments exist in nature. Although the above-mentioned OTU classification was a rough estimation and lacked the precision of actual characteristics, the results of the present study deepen our understanding of the influence of MnO_2_ on the microbial community. To the best of our knowledge, the functional classification analysis that compared microbial communities, which were enriched under different environmental conditions, was applied in the present study for the first time and may be applicable to investigations on bacteria that are tolerant and intolerant of other metal oxides.

Among the 48 MnOB isolated in the present study, the genus *Pseudomonas* had the highest Mn(II) oxidation capacity. *P. putida* GB-1, which has been intensively examined as a MnOB model, possesses a high Mn(II) oxidation ability. Although the 6 MnOB belonged to the genus *Pseudomonas*, their ability to perform Mn(II) oxidation differed. Mn(II) oxidation occurs at an early stage of cultivation (Okazaki *et al.*, 1997). Bio-MnO_x_ production was observed in a batch experiment for the isolation of MnOB after nearly one week; the duration required for strain GB-1 was only 2 days (Geszvain *et al.*, 2011). Mn(II) oxidation by strain GB-1 was observed during MCO production associated with the genes *mcoA* and *mnxG*, and the production of animal heme peroxidase associated with the *mopA* gene (Geszvain *et al.*, 2016). The number of these genes differ, even among strains of *P. putida* (Geszvain *et al.*, 2013), which suggests that Mn(II) oxidation capacity depends on the number of bacteria present and also that several MnOB with different Mn(II) oxidation abilities have been observed in the genus *Pseudomonas*.

In the family *Comamonadaceae*, we isolated 35 MnOB from the genera *Acidovorax*, *Comamonas*, and *Delftia*. The Mn(II) oxidation ability of *Variovorax* sp., which was detected in a ferromanganese deposit by 16S rRNA gene sequencing, has not yet been reported (Northup *et al.*, 2003), and this may be because of the difficulties associated with confirming it as MnOB since the Mn(II) oxidation ability of the genus *Variovorax* is very weak. The GenBank database shows that among the *moxA*, *mofA*, *mnxG*, and *mopA* genes, which are considered to be responsible for Mn(II) oxidation, only *moxA* was conserved as a common gene for the genera *Comamonas*, *Acidovorax*, and *Delftia* (Table S4). Although *moxA* was discovered as a gene that supported Mn(II) oxidation in *Pedomicrobium* sp. (Ridge *et al.*, 2007), the *cumA* of‍ ‍the *moxA* homolog is not involved in Mn(II) oxidation by‍ ‍*P. putida* GB-1 (Geszvain and Tebo, 2010). Furthermore, the *moxA* query cover ratio of *P. putida* GB-1 to *Pedomicrobium* sp. was lower than that of other bacteria belonging to the family *Comamonadaceae*. Although most of the *moxA* homologs may not function as MCO producers, they may be widely conserved in a variety of bacteria for survival in harsh environments, such as that of Cu(II) inhibition. However, *Leptothrix discophora*, a known MnOB (De Vrind *et al.*, 1990) contains the *mofA* gene only, and a mutation analysis revealed that the MCO derived from *mofA* was‍ ‍involved in Mn(II) oxidation (Corstjens *et al.*, 1997; Brouwers *et al.*, 1999).

Phylogenetically diverse MnOB have been discovered and the types of genes associated with Mn(II) oxidation and the number of genes in different MnOB varied. Thus, Mn(II) oxidation potential may differ among MnOB. Even if MnOB are cultured in bioreactors, the levels of Mn(II) oxidation and Bio-MnO_x_ production will remain unclear. To achieve higher Mn(II) oxidation rates in reactors, it is necessary to define the operational methodology for the enrichment of predominant MnOB with high Mn(II) oxidation capacities. In addition, the inhibitory mechanism employed by MnO_2_ towards the growth of specific bacteria requires further investigation.

## Citation

Matsushita, S., Hiroe, T., Kambara, H., Shoiful, A., Aoi, Y., Kindaichi, T., et al. (2020) Anti-bacterial Effects of MnO_2_ on the Enrichment of Manganese-oxidizing Bacteria in Downflow Hanging Sponge Reactors. *Microbes Environ ***35**: ME20052.

https://doi.org/10.1264/jsme2.ME20052

## Supplementary Material

Supplementary Material

## Figures and Tables

**Table 1.** DHS reactor operating conditions

Run	Phase^a^	Day	Mn(II) (mg L^–1^)	COD (mg L^–1^)	HRT (hours)	Objectives
1	1	0–127	5	50	4.25	Negative control for analyzing the effects of MnO_2_
2	1	0–39	5–10	15–50	1.25–4.25	Evaluation of increasing Mn(II) oxidation activity
	2	40–56	5	50	4.25	Effects of loading amount changes
	3	57–127	5	3–6	0.25–0.50	Evaluation of the maximum Mn(II) removal rate
	4	128–198	5	50	4.25	Maintained for biomass sampling
3	1	0–67	0	50	4.25	MnOB enrichment without Mn(II)
	2	68–127	5–10	3–50	0.25–4.25	Evaluation of the maximum Mn(II) removal rate

^a^ Changes in conditions during each phase defined the minimum and maximum values

**Table 2.** Mn-oxidizing ability of isolated bacteria

Phylum	Class	Family	Genus	Number of isolated bacteria	Number of positive reactions in the LBB spot test^a^
*Actinobacteria*	*Actinobacteria*	*Microbacteriaceae*	*Microbacterium*	1	0
		*Nocardiaceae*	*Rhodococcus*	5	1
*Proteobacteria*	*Alphaproteobacteria*	*Rhizobiaceae*	*Ensifer*	2	1
		*Sphingomonadaceae*	*Sphingomonas*	1	1
	*Betaproteobacteria*	*Comamonadaceae*	*Acidovorax*	8	3
			*Comamonas*	41	31
			*Curvibacter*	3	0
			*Delftia*	9	1
		Unclassified	*Mitsuaria*	1	1
	*Gammaproteobacteria*	*Pseudomonadaceae*	*Pseudomonas*	6	6 (3)
		*Xanthomonadaceae*	*Tahibacter*	1	0
			*Stenotrophomonas*	1	0
*Firmicutes*	*Bacilli*	*Bacillaceae*	*Bacillus*	5	3
Total				84	48 (3)

^a^ Parentheses indicate the number of strains with a high Mn(II) oxidation capacity

**Table 3.** Classification of bacteria grown in feed solutions

Group	OTUs	SR1 (day 35)			SR2 (day 35)			SR3.1 (day 35)			SR3.2 (day 73)	
		No. of sequences (Reads)	Ratio (%)^a^		No. of sequences (Reads)	Ratio (%)^a^		No. of sequences (Reads)	Ratio (%)^a^		No. of sequences (Reads)	Ratio (%)^a^
A/B	18	0	0.0		1,158	1.9		1,844	2.9		2,856	4.8
B	18	6,843	10.7		12,644	20.5		6,642	10.5		4,148	6.9
C	272	42,173	65.7		47,922	77.6		54,743	86.6		52,746	88.3
D	5	554	0.9		0	0.0		0	0.0		0	0.0
E	61	14,616	22.8		0	0.0		0	0.0		0	0.0

^a^ Percentage of sequence reads in each group to the total number of sequence reads
