# Supplementary material for: Anti-bacterial Effects of MnO2 on the Enrichment of Manganese-oxidizing Bacteria in Downflow Hanging Sponge Reactors
Source: Microbes Environ. 2020 Sep 19;35(4):ME20052. doi: 10.1264/jsme2.ME20052 (PMC7734401; doi:10.1264/jsme2.ME20052)
Supplement: Supplementary file 1 — Supplementary Material [file 35_20052_s1.pdf]

Supplementary material

Preliminary experiment

Determination of colony-forming units on culture plates with and without MnO_2.

1. Materials and Methods

(1) Biomass sample

Colony forming units (CFUs) of activated sludge, which was collected from an aeration tank in a municipal wastewater treatment plant, were examined. Next, 1.5 mL of sludge was briefly homogenized by ultrasonication for 10 s.

(2) Culture plate

Two types of culture plates were prepared as follows:

(i) Conventional plate medium without abiotic MnO_2: gellan gum (Wako Pure Chem. Co., Osaka, Japan) with a concentration of 25 g L^{-1} containing 0.2 g L^{-1} peptone, 0.05 g L^{-1} yeast extract, and 20 mM HEPES (pH 7.2).

(ii) Specific plate medium with abiotic MnO_2 containing the conventional gellan gum medium and 100 g L^{-1} of MnO_2.

(3) Cultivation

The homogenized sludge was diluted 10^1–10^7 times with PBS buffer (pH 7.4). Each diluted 50-µL sample was spread on the gellan gum plates, after which the plates were incubated at 30°C. After cultivation for 5 days, the CFUs were determined.

(4) Estimation of Mn(II) oxidation ability

To estimate Mn(II) oxidation ability, colonies formed on specific plates containing abiotic MnO_2 were picked up using a toothpick, and each colony was inoculated into a well plate

containing liquid medium (0.2 g L^{-1} peptone, 0.05 g L^{-1} yeast extract, 5 mg Mn(II) L^{-1}, 20 mM HEPES [pH 7.2]). The well plate was shaken at 100 rpm. After cultivating the cells for one month at 30°C, Mn oxide formation was assayed by the LBB method. Colonies with Mn(II) oxidation ability were identified to be MnOB.

2. Results

Table S1 CFU and MnOB in activated sludge

Culture plate	CFU/mL	MnOB/mL
Without MnO_2	$9.4 \times 10^7 \pm 1.1 \times 10^7$	—
With MnO_2	$3.7 \times 10^5 \pm 1.0 \times 10^5$	4.0×10^4

We observed that 3.7×10^5 CFU/mL formed on the MnO_2 plate for the activated sludge, while 9.4×10^7 CFU/mL were observed on the non-MnO_2 plate. Colony formation of bacteria in the activated sludge was inhibited by 99.6% on the MnO_2 plate. Only 0.4% of the bacteria in the activated sludge was MnO_2 tolerant. Of the MnO_2-tolerant colonies, 10.7% were estimated to be MnOB.

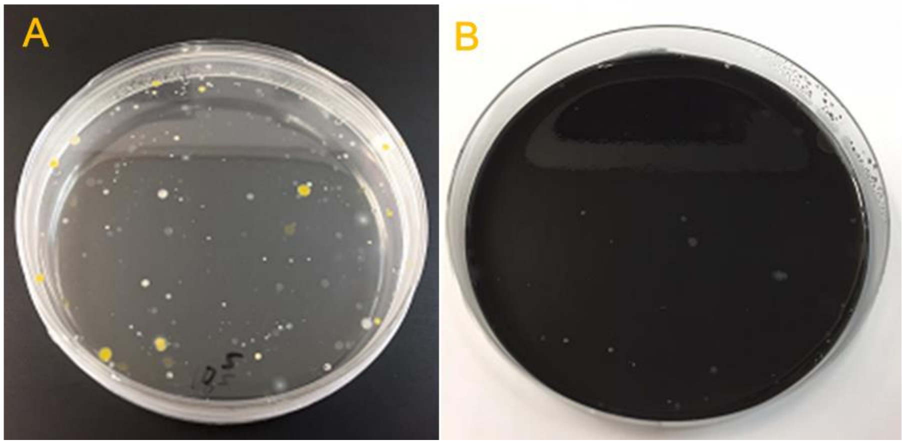

Photo S1 Representative results of CFUs. (A) Plate without MnO_2 at a dilution of 10^5, (B) Plate with MnO_2 at a dilution of 10^3.

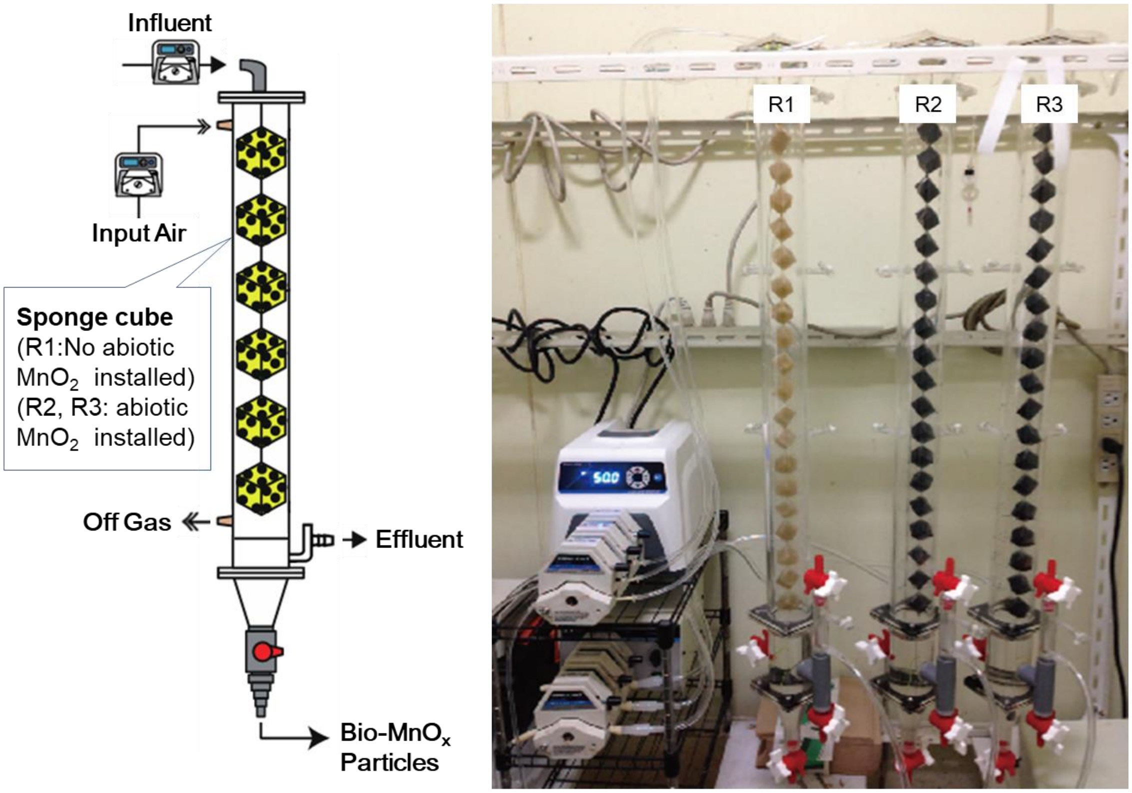

Fig. S1 Schematic diagram (left) and photograph (right) of the downflow hanging sponge reactor used to enrich MnOB for the removal and recovery of minor metals from wastewater. Artificial wastewater containing Mn(II) and air was supplied to the reactor.

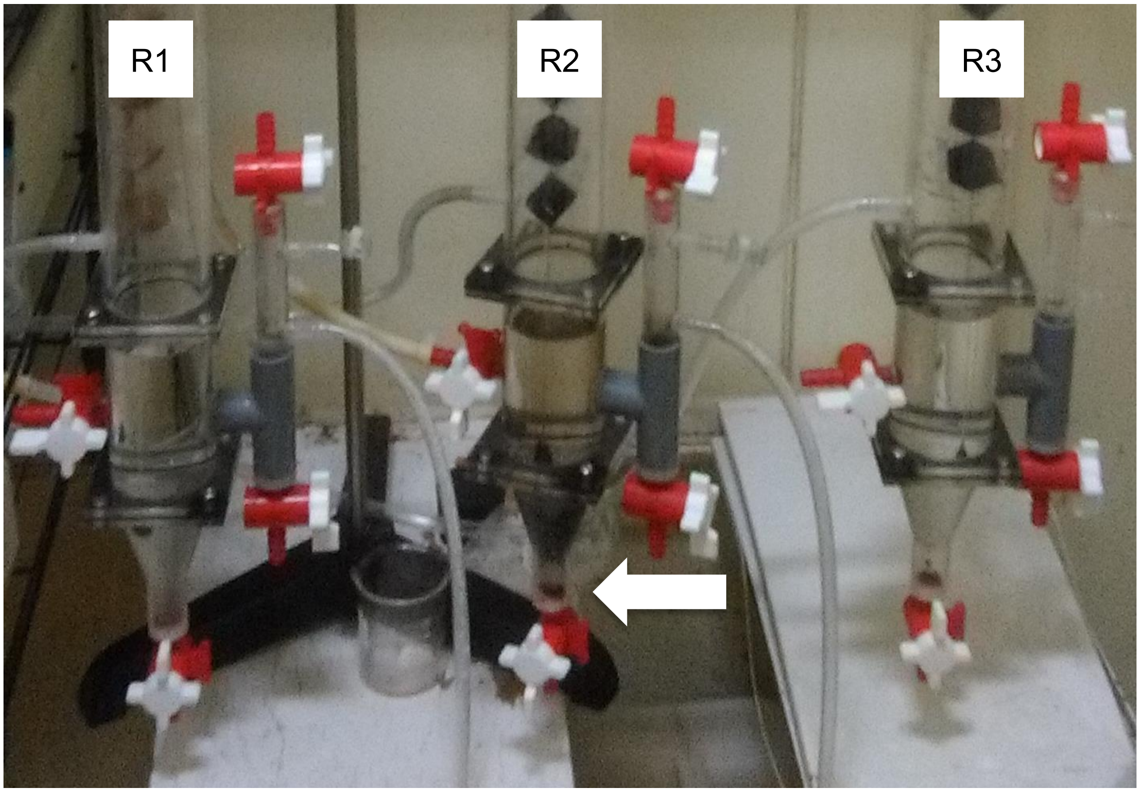

Photo S2 Fine black particles deposited at the bottom were observed in the R2 reactor on day 44 (white arrow).

Table S2 Population size of organisms that are phylogenetically close to MnOB

Family	Genus	SR1 (day 35) No. of sequences (Reads)	SR1 (day 35) Ratio (%)[a]	SR2 (day 35) No. of sequences (Reads)	SR2 (day 35) Ratio (%)[a]	SR3.1 (day 35) No. of sequences (Reads)	SR3.1 (day 35) Ratio (%)[a]	SR3.2 (day 73) No. of sequences (Reads)	SR3.2 (day 73) Ratio (%)[a]	Reference
Nocardiaceae	*Nocardia*	0	0.0	81	0.1	0	0.0	3	0.0	Ghiorse, 1984
	Rhodococcus	1	0.0	1	0.0	2	0.0	0	0.0	Barboza et al., 2015
	Unclassfied	0	0.0	40	0.1	0	0.0	7	0.0	-
Cytophagaceae	*Cytophaga*	50	0.1	11	0.0	28	0.0	22	0.0	Ghiorse, 1984
	Unclassfied	2,541	3.2	5,791	8.0	2,897	3.8	976	1.3	-
Bacillaceae	*Bacillus*	32	0.0	8	0.0	2	0.0	1	0.0	Nealson and Ford, 1980
	Unclassfied	1	0.0	0	0.0	0	0.0	0	0.0	-
Hyphomicrobiaceae	*Hyphomicrobium*	206	0.3	299	0.4	258	0.3	100	0.1	Tyler et al., 1967
	Pedomicrobium	53	0.1	34	0.0	64	0.1	37	0.1	Gebers 1981
	Unclassfied	20	0.0	10	0.0	75	0.1	34	0.0	-
Rhizobiaceae	*Agrobacterium*	0	0.0	2	0.0	15	0.0	12	0.0	Santelli et al., 2010
	Ensifer	0	0.0	0	0.0	0	0.0	0	0.0	Piazza et al., 2019
	Unclassfied	76	0.1	122	0.2	542	0.7	402	0.5	-
Rhodobacteraceae	*Rhodobacter*	229	0.3	111	0.2	292	0.4	136	0.2	Anderson et al., 2009
	Unclassfied	22	0.0	0	0.0	12	0.0	7	0.0	-
Sphingomonadaceae	*Sphingomonas*	98	0.1	26	0.0	150	0.2	787	1.1	Francis et al., 2001
	Unclassfied	473	0.6	202	0.3	495	0.7	1,073	1.5	-
Burkholderiaceae	*Mitsuaria*	0	0.0	0	0.0	0	0.0	0	0.0	Current study
	Unclassfied	0	0.0	26	0.0	0	0.0	3	0.0	-
Comamonadaceae	*Acidovorax*	4	0.0	8	0.0	9	0.0	12	0.0	Tsuji et al., 2017
	Comamonas	2	0.0	3	0.0	4	0.0	3	0.0	Tsuji et al., 2017
	Delftia	4	0.0	5	0.0	5	0.0	0	0.0	Current study
	Leptothrix	0	0.0	1	0.0	7	0.0	0	0.0	Adams and Ghiorse, 1985
	Unclassfied	3,973	5.0	3,348	4.6	4,828	6.3	2,867	3.9	-
Enterobacteriaceae	*Enterobacter*	109	0.1	3	0.0	2	0.0	6	0.0	Ghiorse, 1984
	Unclassfied	6	0.0	2	0.0	2	0.0	4	0.0	-
Pseudomonadaceae	*Pseudomonas*	1,239	1.6	4,837	6.7	140	0.2	1,249	1.7	Okazaki et al., 1997
	Unclassfied	3	0.0	2	0.0	4	0.0	0	0.0	-
Number of sequences phylogenetically close to MnOB		9,142	11.6	14,973	20.5	9,833	12.9	7,741	10.6	
Sequence numbers for all bacteria		79,067	100.0	72,513	100.0	76,060	100.0	73,197	100.0	

[a]Percentage of sequence reads of each group to the number of whole sequence reads

References

Adams, L.F., and Ghiorse, W.C. (1985) Influence of manganese on growth of a sheathless strain of *Leptothrix discophora*. Appl Environ Microbiol **49**: 556–562.

Anderson, C.R., Johnson, H.A., Caputo, N., Davis, R.E., Torpey, J.W., and Tebo, B.M. (2009) Mn(II) oxidation is catalyzed by heme peroxidases in "*Aurantimonas manganoxydans*" strain SI85-9A1 and *Erythrobacter* sp. strain SD-21. Appl Environ Microbiol **75**: 4130–4138. https://doi.org/10.1128/AEM.02890-08.

Barboza, N.R., Amorim, S.S., Santos, P.A., Reis, F.D., Cordeiro, M.M., Guerra-sá, et al. (2015) Indirect manganese removal by *Stenotrophomonas* sp. and *Lysinibacillus* sp. isolated from Brazilian mine water. Biomed Res Int **2015**: 18–20.

Francis, C. a, Co, E.M., and Tebo, B.M. (2001) Enzymatic manganese (II) oxidation by a marine alpha-proteobacterium. Appl Environ Microbiol **67**: 4024–4029.

Gebers, R. (1981) Enrichment, isolation, and emended description of *Pedomicrobium ferrugineum* Aristovskaya and *Pedomicrobium manganicum* Aristovskaya. Int J Syst Bacteriol **31**: 302–316.

Ghiorse, W.C. (1984) Biology of iron- and manganese-depositing bacteria. Annu Rev Microbiol **38**: 515–550.

Nealson, K.H., and Ford, J. (1980) Surface enhancement of bacterial manganese oxidation: Implications for aquatic environments. Geomicrobiol J **2**: 21–37.

Okazaki, M., Sugita, T., Shimizu, M., Ohode, Y., Iwamoto, K., deVrind-deJong, E.W., et al. (1997) Partial purification and characterization of manganese-oxidizing factors of *Pseudomonas putida* GB-1. Appl Environ Microbiol **63**: 4793–4799.

Piazza, A., Casalini, L.C., Pacini, V.A., Sanguinetti, G., Ottado, J., Gottig, N. (2019) Environmental bacteria involved in manganese(II) oxidation and removal from groundwater. Front Microbiol **10**: 1–13.

Santelli, C.M., Pfister, D.H., Lazarus, D., Sun, L., Burgos, W.D., and Hansel, C.M. (2010) Promotion of Mn(II) oxidation and remediation of coal mine drainage in passive treatment systems by diverse fungal and bacterial communities. Appl Environ Microbiol **76**: 4871–4875. https://doi.10.1128/AEM.03029-09.

Tsuji, K., Asayama, T., Shiraki, N., Inoue, S., Okuda, E., Hayashi, C., et al. (2017) Mn accumulation in a submerged plant *Egeria densa* (Hydrocharitaceae) is mediated by epiphytic bacteria. Plant Cell Environ **40**: 1163–1173.

Tyler, P.A., and Marshall, K.C. (1967) Microbial oxidation of manganese in hydro-electric pipelines. Antonie Van Leeuwenhoek **33**: 171–183.

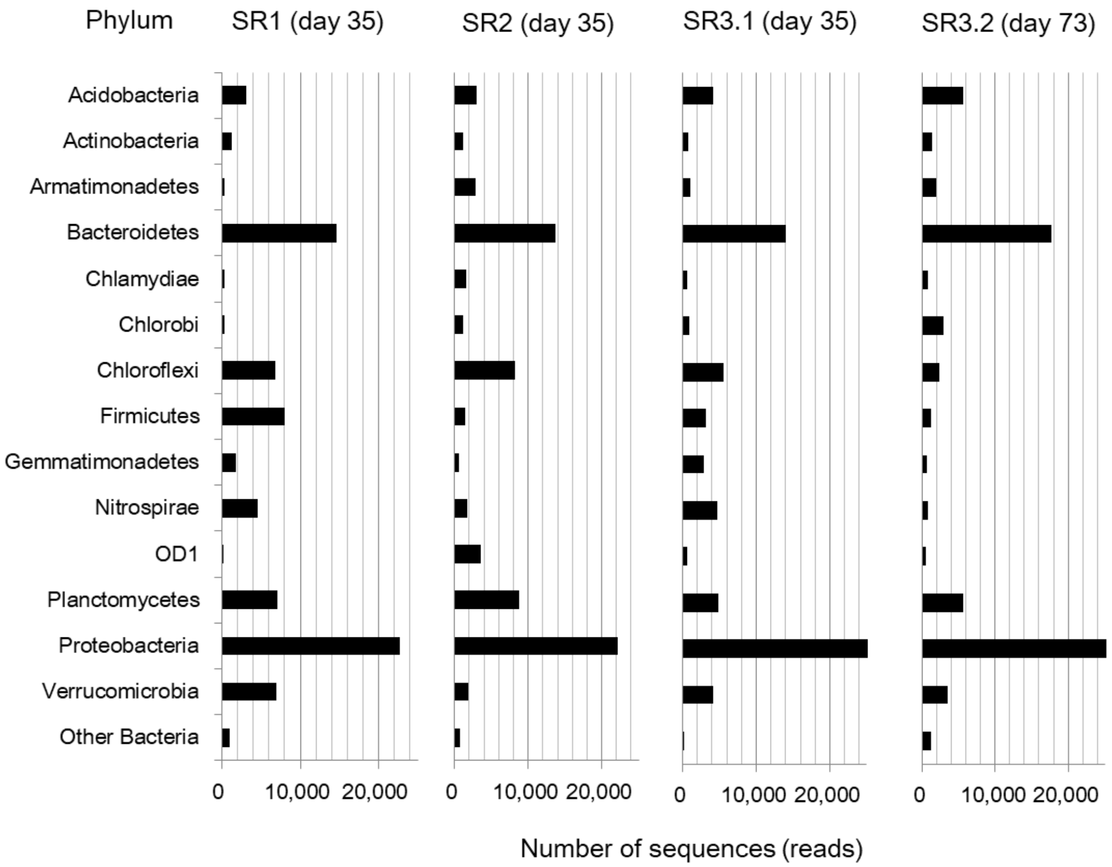

Fig. S2 Sequence number comparison of different phylum levels in the microbial community.

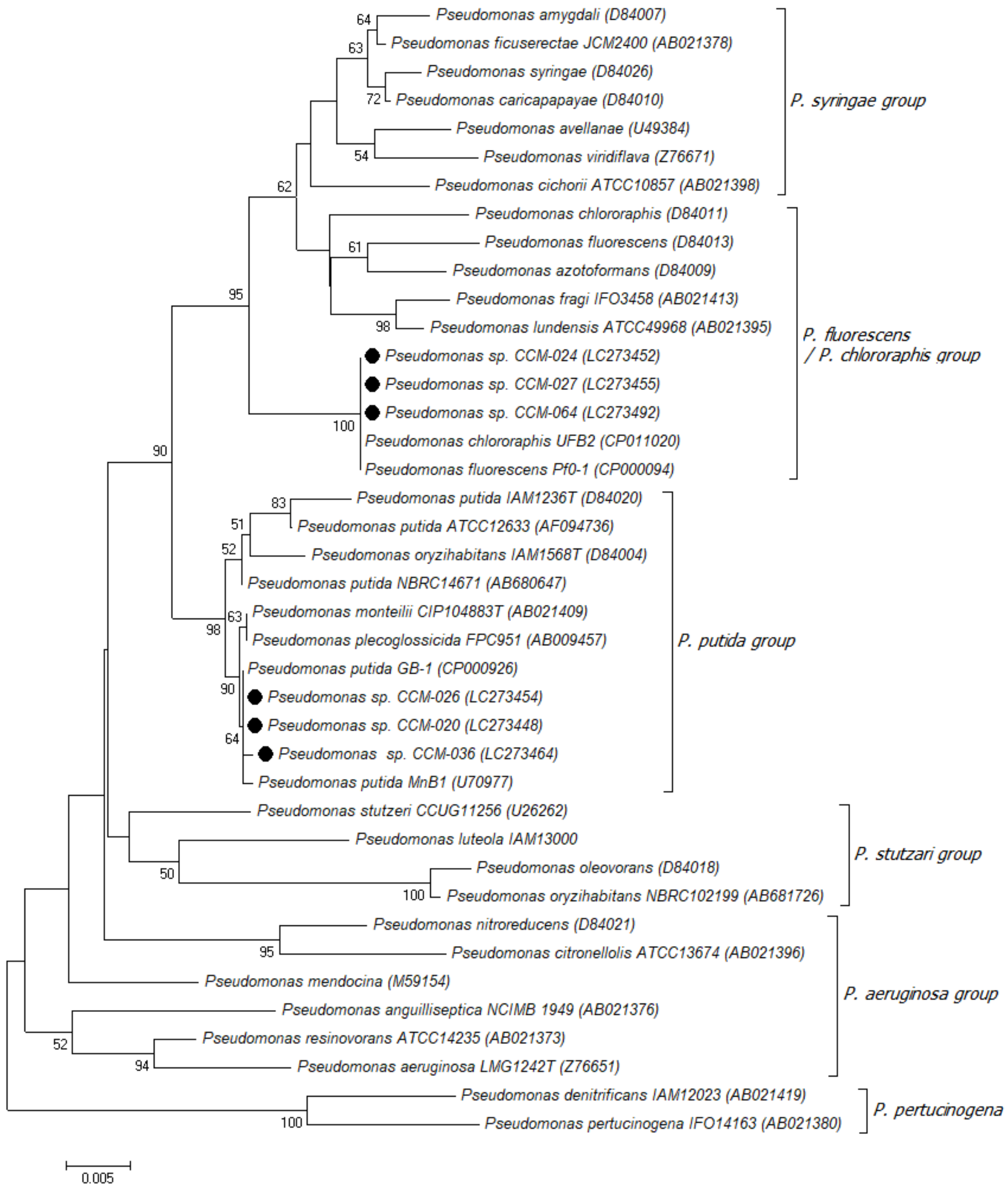

Fig. S3 Neighbor-joining phylogenetic tree showing the isolated *Pseudomonas* genera. The percentage of bootstrap support (> 50%) from 1,000 replicates is indicated at the branch points.

Table S3 The selected OTUs and classification based on a comparison of microbial communities between Runs 1, 2, and 3.

OTU No.	Phylum	Class	Order	Family	Genus	SR1 (Reads)	SR1 (%)	SR2 (Reads)	SR2 (%)	SR3.1 (Reads)	SR3.1 (%)	SR3.2 (Reads)	SR3.2 (%)	Group
731	Acidobacteria	[Chloracidobacteria]	RB41	Ellin6075	.	0	0.00	127	0.21	119	0.19	99	0.17	C
2542	Acidobacteria	[Chloracidobacteria]	RB41	Ellin6075	.	0	0.00	0	0.00	0	0.00	347	0.58	C
3491	Acidobacteria	[Chloracidobacteria]	RB41	Ellin6075	.	83	0.13	557	0.90	638	1.01	1069	1.79	C
3983	Acidobacteria	[Chloracidobacteria]	RB41	Ellin6075	.	0	0.00	0	0.00	544	0.86	222	0.37	C
4570	Acidobacteria	[Chloracidobacteria]	RB41	Ellin6075	.	480	0.75	93	0.15	340	0.54	580	0.97	C
5794	Acidobacteria	[Chloracidobacteria]	RB41	Ellin6075	.	0	0.00	0	0.00	0	0.00	880	1.47	C
6136	Acidobacteria	[Chloracidobacteria]	RB41	Ellin6075	.	0	0.00	0	0.00	0	0.00	83	0.14	C
7856	Acidobacteria	[Chloracidobacteria]	RB41	Ellin6075	.	150	0.23	137	0.22	0	0.00	0	0.00	C
8998	Acidobacteria	Acidobacteria-6	iii1-15	mb2424	.	0	0.00	112	0.18	0	0.00	0	0.00	C
894	Acidobacteria	Holophagae	Holophagales	Holophagaceae	.	0	0.00	0	0.00	669	1.06	0	0.00	C
437	Acidobacteria	Solibacteres	Solibacterales	.	.	0	0.00	0	0.00	0	0.00	75	0.13	C
3751	Acidobacteria	Solibacteres	Solibacterales	.	.	1185	1.85	852	1.38	559	0.88	0	0.00	C
4633	Acidobacteria	Solibacteres	Solibacterales	.	.	0	0.00	0	0.00	0	0.00	91	0.15	C
5351	Acidobacteria	Solibacteres	Solibacterales	.	.	379	0.59	0	0.00	714	1.13	958	1.60	C
10605	Acidobacteria	Solibacteres	Solibacterales	.	.	0	0.00	426	0.69	0	0.00	213	0.36	C
9765	Acidobacteria	Solibacteres	Solibacterales	Solibacteraceae	.	0	0.00	211	0.34	98	0.15	100	0.17	C
2851	Actinobacteria	Acidimicrobiia	Acidimicrobiales	C111	.	0	0.00	108	0.17	0	0.00	0	0.00	C
6256	Actinobacteria	Acidimicrobiia	Acidimicrobiales	JdFBGBact	.	0	0.00	138	0.22	0	0.00	0	0.00	C
1047	Actinobacteria	Actinobacteria	Actinomycetales	.	.	97	0.15	0	0.00	0	0.00	0	0.00	E
7463	Actinobacteria	Actinobacteria	Actinomycetales	Gordoniaceae	Gordonia	98	0.15	0	0.00	0	0.00	490	0.82	C
10602	Actinobacteria	Actinobacteria	Actinomycetales	Microbacteriaceae	Leucobacter	0	0.00	0	0.00	288	0.46	0	0.00	C
6646	Actinobacteria	Actinobacteria	Actinomycetales	Microbacteriaceae	Microbacterium	115	0.18	0	0.00	0	0.00	176	0.29	C
7304	Actinobacteria	Actinobacteria	Actinomycetales	Mycobacteriaceae	Mycobacterium	112	0.17	221	0.36	0	0.00	342	0.57	C
6456	Actinobacteria	Actinobacteria	Actinomycetales	Nocardiaceae	Nocardia	0	0.00	81	0.13	0	0.00	0	0.00	A/B
4299	Actinobacteria	Actinobacteria	Actinomycetales	Nocardioidaceae	Nocardioides	0	0.00	103	0.17	0	0.00	0	0.00	C

10578	Actinobacteria	Actinobacteria	Actinomycetales	Pseudonocardiaceae	Pseudonocardia	88	0.14	0	0.00	0	0.00	0	0.00	E
2874	Armatimonadetes	[Fimbriimonadia]	[Fimbriimonadales]	[Fimbriimonadaceae]	.	131	0.20	0	0.00	0	0.00	0	0.00	E
4963	Armatimonadetes	[Fimbriimonadia]	[Fimbriimonadales]	[Fimbriimonadaceae]	Fimbriimonas	0	0.00	194	0.31	0	0.00	0	0.00	C
5502	Armatimonadetes	[Fimbriimonadia]	[Fimbriimonadales]	[Fimbriimonadaceae]	Fimbriimonas	111	0.17	0	0.00	0	0.00	0	0.00	E
7011	Armatimonadetes	[Fimbriimonadia]	[Fimbriimonadales]	[Fimbriimonadaceae]	Fimbriimonas	0	0.00	0	0.00	255	0.40	0	0.00	C
8649	Armatimonadetes	[Fimbriimonadia]	[Fimbriimonadales]	[Fimbriimonadaceae]	Fimbriimonas	0	0.00	0	0.00	166	0.26	0	0.00	C
839	Armatimonadetes	Armatimonadia	Armatimonadales	Armatimonadaceae	.	0	0.00	250	0.41	0	0.00	0	0.00	C
8101	Armatimonadetes	Armatimonadia	Armatimonadales	Armatimonadaceae	.	0	0.00	113	0.18	0	0.00	0	0.00	C
2610	Armatimonadetes	Chthonomonadetes	Chthonomonadales	Chthonomonadaceae	.	0	0.00	158	0.26	0	0.00	0	0.00	C
770	Armatimonadetes	Chthonomonadetes	Chthonomonadales	Chthonomonadaceae	Chthonomonas	0	0.00	661	1.07	0	0.00	0	0.00	C
771	Armatimonadetes	Chthonomonadetes	SJA-22	.	.	0	0.00	1094	1.77	0	0.00	525	0.88	C
1931	Armatimonadetes	Chthonomonadetes	SJA-22	.	.	0	0.00	179	0.29	0	0.00	741	1.24	C
2383	Armatimonadetes	Chthonomonadetes	SJA-22	.	.	0	0.00	0	0.00	134	0.21	0	0.00	C
6858	Armatimonadetes	Chthonomonadetes	SJA-22	.	.	0	0.00	0	0.00	280	0.44	223	0.37	C
5147	Bacteroidetes	[Saprospirae]	[Saprospirales]	.	.	0	0.00	0	0.00	0	0.00	126	0.21	C
1365	Bacteroidetes	[Saprospirae]	[Saprospirales]	Chitinophagaceae	.	0	0.00	149	0.24	0	0.00	0	0.00	C
1838	Bacteroidetes	[Saprospirae]	[Saprospirales]	Chitinophagaceae	.	0	0.00	0	0.00	258	0.41	0	0.00	C
2023	Bacteroidetes	[Saprospirae]	[Saprospirales]	Chitinophagaceae	.	5016	7.81	0	0.00	0	0.00	0	0.00	E
2223	Bacteroidetes	[Saprospirae]	[Saprospirales]	Chitinophagaceae	.	176	0.27	0	0.00	0	0.00	0	0.00	E
2779	Bacteroidetes	[Saprospirae]	[Saprospirales]	Chitinophagaceae	.	959	1.49	0	0.00	429	0.68	119	0.20	C
3062	Bacteroidetes	[Saprospirae]	[Saprospirales]	Chitinophagaceae	.	209	0.33	138	0.22	3690	5.84	10358	17.34	C
3378	Bacteroidetes	[Saprospirae]	[Saprospirales]	Chitinophagaceae	.	86	0.13	0	0.00	0	0.00	0	0.00	E
3455	Bacteroidetes	[Saprospirae]	[Saprospirales]	Chitinophagaceae	.	0	0.00	0	0.00	93	0.15	0	0.00	C
4396	Bacteroidetes	[Saprospirae]	[Saprospirales]	Chitinophagaceae	.	0	0.00	0	0.00	154	0.24	343	0.57	C
6252	Bacteroidetes	[Saprospirae]	[Saprospirales]	Chitinophagaceae	.	157	0.24	0	0.00	0	0.00	0	0.00	E
7736	Bacteroidetes	[Saprospirae]	[Saprospirales]	Chitinophagaceae	.	0	0.00	0	0.00	190	0.30	0	0.00	C
8588	Bacteroidetes	[Saprospirae]	[Saprospirales]	Chitinophagaceae	.	112	0.17	0	0.00	151	0.24	0	0.00	C
9885	Bacteroidetes	[Saprospirae]	[Saprospirales]	Chitinophagaceae	.	0	0.00	0	0.00	0	0.00	355	0.59	C

9918	Bacteroidetes	[Saprospirae]	[Saprospirales]	Chitinophagaceae	.	246	0.38	0	0.00	315	0.50	0	0.00	C
10275	Bacteroidetes	[Saprospirae]	[Saprospirales]	Chitinophagaceae	.	0	0.00	0	0.00	0	0.00	264	0.44	C
10294	Bacteroidetes	[Saprospirae]	[Saprospirales]	Chitinophagaceae	.	851	1.33	133	0.22	2002	3.17	743	1.24	C
10297	Bacteroidetes	[Saprospirae]	[Saprospirales]	Chitinophagaceae	.	95	0.15	0	0.00	167	0.26	0	0.00	C
10845	Bacteroidetes	[Saprospirae]	[Saprospirales]	Chitinophagaceae	.	311	0.48	0	0.00	315	0.50	78	0.13	C
5676	Bacteroidetes	[Saprospirae]	[Saprospirales]	Chitinophagaceae	Flavihumibacter	130	0.20	0	0.00	0	0.00	0	0.00	E
9450	Bacteroidetes	[Saprospirae]	[Saprospirales]	Chitinophagaceae	Flavihumibacter	0	0.00	0	0.00	211	0.33	0	0.00	C
8229	Bacteroidetes	[Saprospirae]	[Saprospirales]	Chitinophagaceae	Sediminibacterium	192	0.30	0	0.00	0	0.00	0	0.00	E
10913	Bacteroidetes	[Saprospirae]	[Saprospirales]	Chitinophagaceae	Sediminibacterium	0	0.00	0	0.00	111	0.18	0	0.00	C
769	Bacteroidetes	[Saprospirae]	[Saprospirales]	Saprospiraceae	.	0	0.00	806	1.31	0	0.00	0	0.00	C
6095	Bacteroidetes	Bacteroidia	Bacteroidales	Rikenellaceae	Blvii28	0	0.00	0	0.00	495	0.78	0	0.00	C
2293	Bacteroidetes	Cytophagia	Cytophagales	.	.	102	0.16	0	0.00	0	0.00	0	0.00	E
2982	Bacteroidetes	Cytophagia	Cytophagales	Cytophagaceae	.	330	0.51	1075	1.74	0	0.00	465	0.78	B
3892	Bacteroidetes	Cytophagia	Cytophagales	Cytophagaceae	.	290	0.45	1944	3.15	0	0.00	0	0.00	B
4391	Bacteroidetes	Cytophagia	Cytophagales	Cytophagaceae	.	0	0.00	608	0.99	0	0.00	211	0.35	A/B
4571	Bacteroidetes	Cytophagia	Cytophagales	Cytophagaceae	.	880	1.37	283	0.46	0	0.00	0	0.00	B
4573	Bacteroidetes	Cytophagia	Cytophagales	Cytophagaceae	.	355	0.55	1182	1.91	1494	2.36	0	0.00	B
6867	Bacteroidetes	Cytophagia	Cytophagales	Cytophagaceae	.	190	0.30	0	0.00	0	0.00	0	0.00	D
9329	Bacteroidetes	Cytophagia	Cytophagales	Cytophagaceae	.	0	0.00	0	0.00	517	0.82	0	0.00	A/B
9581	Bacteroidetes	Cytophagia	Cytophagales	Cytophagaceae	.	89	0.14	0	0.00	538	0.85	0	0.00	B
10342	Bacteroidetes	Cytophagia	Cytophagales	Cytophagaceae	.	0	0.00	151	0.24	0	0.00	0	0.00	A/B
10529	Bacteroidetes	Cytophagia	Cytophagales	Cytophagaceae	.	138	0.22	329	0.53	150	0.24	95	0.16	B
1464	Bacteroidetes	Cytophagia	Cytophagales	Cytophagaceae	Flectobacillus	0	0.00	352	0.57	0	0.00	82	0.14	C
5912	Bacteroidetes	Cytophagia	Cytophagales	Cytophagaceae	Runella	0	0.00	0	0.00	0	0.00	3083	5.16	C
7497	Bacteroidetes	Flavobacteriia	Flavobacteriales	[Weeksellaceae]	Chryseobacterium	0	0.00	0	0.00	0	0.00	81	0.14	C
4406	Bacteroidetes	Flavobacteriia	Flavobacteriales	Cryomorphaceae	.	0	0.00	0	0.00	131	0.21	0	0.00	C
531	Bacteroidetes	Sphingobacteriia	Sphingobacteriales	.	.	818	1.27	0	0.00	165	0.26	0	0.00	C
1207	Bacteroidetes	Sphingobacteriia	Sphingobacteriales	.	.	0	0.00	211	0.34	0	0.00	0	0.00	C
4121	Bacteroidetes	Sphingobacteriia	Sphingobacteriales	.	.	0	0.00	0	0.00	0	0.00	120	0.20	C

5771	Bacteroidetes	Sphingobacteriia	Sphingobacteriales	.	.	92	0.14	123	0.20	249	0.39	0	0.00	C
6029	Bacteroidetes	Sphingobacteriia	Sphingobacteriales	.	.	0	0.00	0	0.00	149	0.24	0	0.00	C
6176	Bacteroidetes	Sphingobacteriia	Sphingobacteriales	.	.	159	0.25	0	0.00	0	0.00	0	0.00	E
10882	Bacteroidetes	Sphingobacteriia	Sphingobacteriales	.	.	0	0.00	0	0.00	95	0.15	0	0.00	C
11096	Bacteroidetes	Sphingobacteriia	Sphingobacteriales	.	.	92	0.14	0	0.00	0	0.00	0	0.00	E
11263	Bacteroidetes	Sphingobacteriia	Sphingobacteriales	.	.	630	0.98	5343	8.66	364	0.58	0	0.00	C
3644	Bacteroidetes	Sphingobacteriia	Sphingobacteriales	Sphingobacteriaceae	.	106	0.17	0	0.00	0	0.00	0	0.00	E
1686	Chlamydiae	Chlamydiia	Chlamydiales	.	.	0	0.00	156	0.25	0	0.00	0	0.00	C
2426	Chlamydiae	Chlamydiia	Chlamydiales	.	.	0	0.00	0	0.00	0	0.00	109	0.18	C
6461	Chlamydiae	Chlamydiia	Chlamydiales	.	.	0	0.00	0	0.00	91	0.14	0	0.00	C
7070	Chlamydiae	Chlamydiia	Chlamydiales	.	.	0	0.00	337	0.55	0	0.00	0	0.00	C
7209	Chlamydiae	Chlamydiia	Chlamydiales	.	.	0	0.00	365	0.59	0	0.00	0	0.00	C
740	Chlamydiae	Chlamydiia	Chlamydiales	Parachlamydiaceae	Parachlamydia	0	0.00	0	0.00	122	0.19	158	0.26	C
8979	Chlamydiae	Chlamydiia	Chlamydiales	Parachlamydiaceae	Parachlamydia	0	0.00	243	0.39	0	0.00	0	0.00	C
1757	Chlamydiae	Chlamydiia	Chlamydiales	Rhabdochlamydiaceae	Candidatus Rhabdochlamydia	118	0.18	0	0.00	0	0.00	0	0.00	E
3533	Chlamydiae	Chlamydiia	Chlamydiales	Simkaniaceae	.	0	0.00	0	0.00	91	0.14	0	0.00	C
407	Chlorobi	OPB56	.	.	.	0	0.00	233	0.38	0	0.00	0	0.00	C
1197	Chlorobi	OPB56	.	.	.	0	0.00	0	0.00	166	0.26	0	0.00	C
3532	Chlorobi	OPB56	.	.	.	0	0.00	518	0.84	0	0.00	0	0.00	C
4956	Chlorobi	OPB56	.	.	.	91	0.14	0	0.00	0	0.00	0	0.00	E
4974	Chlorobi	OPB56	.	.	.	0	0.00	0	0.00	0	0.00	2295	3.84	C
3163	Chlorobi	SJA-28	.	.	.	0	0.00	0	0.00	87	0.14	0	0.00	C
4478	Chlorobi	SJA-28	.	.	.	0	0.00	90	0.15	106	0.17	288	0.48	C
6319	Chlorobi	SJA-28	.	.	.	0	0.00	178	0.29	371	0.59	169	0.28	C
6849	Chloroflexi	Anaerolineae	.	.	.	0	0.00	0	0.00	0	0.00	100	0.17	C
7196	Chloroflexi	Anaerolineae	.	.	.	128	0.20	0	0.00	0	0.00	0	0.00	E
6664	Chloroflexi	Anaerolineae	Anaerolineales	Anaerolinaceae	.	0	0.00	0	0.00	169	0.27	0	0.00	C
8045	Chloroflexi	Anaerolineae	Anaerolineales	Anaerolinaceae	.	0	0.00	0	0.00	111	0.18	273	0.46	C
8605	Chloroflexi	Anaerolineae	Caldilineales	Caldilineaceae	.	202	0.31	0	0.00	0	0.00	0	0.00	E

9414	Chloroflexi	Anaerolineae	Caldilineales	Caldilineaceae	.	1731	2.70	3179	5.15	2617	4.14	803	1.34	C
1347	Chloroflexi	Anaerolineae	Caldilineales	Caldilineaceae	Caldilinea	1246	1.94	184	0.30	571	0.90	0	0.00	C
5509	Chloroflexi	Anaerolineae	Caldilineales	Caldilineaceae	Caldilinea	377	0.59	131	0.21	116	0.18	0	0.00	C
6251	Chloroflexi	Anaerolineae	Caldilineales	Caldilineaceae	Caldilinea	86	0.13	0	0.00	0	0.00	0	0.00	E
11092	Chloroflexi	Anaerolineae	envOPS12	.	.	87	0.14	0	0.00	108	0.17	136	0.23	C
1358	Chloroflexi	Anaerolineae	SBR1031	A4b	.	0	0.00	172	0.28	0	0.00	0	0.00	C
3497	Chloroflexi	Anaerolineae	SBR1031	A4b	.	0	0.00	86	0.14	0	0.00	0	0.00	C
1610	Chloroflexi	Anaerolineae	SBR1031	oc28	.	0	0.00	721	1.17	0	0.00	0	0.00	C
5352	Chloroflexi	Anaerolineae	SHA-20	.	.	0	0.00	0	0.00	93	0.15	0	0.00	C
6882	Chloroflexi	Anaerolineae	WCHB1-50	.	.	0	0.00	96	0.16	0	0.00	0	0.00	C
2401	Chloroflexi	Chloroflexi	[Roseiflexales]	[Kouleothrixaceae]	Kouleothrix	514	0.80	280	0.45	567	0.90	266	0.45	C
999	Chloroflexi	Thermomicrobia	JG30-KF-CM45	.	.	1021	1.59	0	0.00	372	0.59	0	0.00	C
1893	Chloroflexi	Thermomicrobia	JG30-KF-CM45	.	.	0	0.00	193	0.31	91	0.14	0	0.00	C
3199	Chloroflexi	Thermomicrobia	JG30-KF-CM45	.	.	0	0.00	117	0.19	0	0.00	0	0.00	C
8519	Chloroflexi	Thermomicrobia	JG30-KF-CM45	.	.	150	0.23	0	0.00	0	0.00	0	0.00	E
7488	Chloroflexi	TK17	mle1-48	.	.	0	0.00	120	0.19	0	0.00	0	0.00	C
9000	Chloroflexi	TK17	mle1-48	.	.	394	0.61	2220	3.60	0	0.00	99	0.17	C
829	Cyanobacteria	Chloroplast	Stramenopiles	.	.	81	0.13	0	0.00	0	0.00	0	0.00	E
175	FCPU426	.	.	.	.	0	0.00	0	0.00	0	0.00	80	0.13	C
6913	Firmicutes	Bacilli	Turicibacterales	Turicibacteraceae	Turicibacter	83	0.13	0	0.00	0	0.00	0	0.00	E
4511	Firmicutes	Clostridia	Clostridiales	.	.	0	0.00	0	0.00	149	0.24	0	0.00	C
2434	Firmicutes	Clostridia	Clostridiales	[Acidaminobacteraceae]	Acidaminobacter	0	0.00	0	0.00	113	0.18	0	0.00	C
5767	Firmicutes	Clostridia	Clostridiales	Clostridiaceae	.	148	0.23	0	0.00	0	0.00	0	0.00	E
1472	Firmicutes	Clostridia	Clostridiales	Clostridiaceae	Clostridium	5837	9.09	907	1.47	2153	3.41	888	1.49	C
3090	Firmicutes	Clostridia	Clostridiales	Clostridiaceae	Clostridium	435	0.68	264	0.43	230	0.36	0	0.00	C
2114	Firmicutes	Clostridia	Clostridiales	Peptostreptococcaceae	.	990	1.54	87	0.14	240	0.38	0	0.00	C
2912	Gemmatimonadetes	Gemm-1	.	.	.	0	0.00	75	0.12	0	0.00	236	0.39	C
2043	Gemmatimonadetes	Gemmatimonadetes	.	.	.	97	0.15	0	0.00	0	0.00	0	0.00	E
9847	Gemmatimonadetes	Gemmatimonadetes	.	.	.	1006	1.57	394	0.64	1313	2.08	111	0.19	C

4247	Gemmatimonadetes	Gemmatimonadetes	Gemmatimonadales	Gemmatimonadaceae	Gemmatimonas	108	0.17	0	0.00	0	0.00	0	0.00	E
10254	Gemmatimonadetes	Gemmatimonadetes	Gemmatimonadales	Gemmatimonadaceae	Gemmatimonas	105	0.16	0	0.00	0	0.00	0	0.00	E
11002	Gemmatimonadetes	Gemmatimonadetes	Gemmatimonadales	Gemmatimonadaceae	Gemmatimonas	383	0.60	0	0.00	1452	2.30	147	0.25	C
7831	Nitrospirae	Nitrospira	Nitrospirales	Nitrospiraceae	Nitrospira	270	0.42	387	0.63	0	0.00	615	1.03	C
8242	Nitrospirae	Nitrospira	Nitrospirales	Nitrospiraceae	Nitrospira	4169	6.50	1236	2.00	4593	7.26	160	0.27	C
767	OD1	SM2F11	.	.	.	0	0.00	1523	2.47	0	0.00	117	0.20	C
6186	OD1	SM2F11	.	.	.	0	0.00	1039	1.68	0	0.00	0	0.00	C
7316	OD1	SM2F11	.	.	.	0	0.00	106	0.17	0	0.00	0	0.00	C
10955	OD1	SM2F11	.	.	.	0	0.00	0	0.00	397	0.63	0	0.00	C
1113	OD1	ZB2	.	.	.	0	0.00	0	0.00	0	0.00	150	0.25	C
1470	OD1	ZB2	.	.	.	0	0.00	0	0.00	174	0.28	0	0.00	C
3384	OD1	ZB2	.	.	.	0	0.00	160	0.26	0	0.00	0	0.00	C
6816	OD1	ZB2	.	.	.	0	0.00	493	0.80	0	0.00	0	0.00	C
379	Planctomycetes	BD7-11	.	.	.	0	0.00	1068	1.73	0	0.00	0	0.00	C
9608	Planctomycetes	OM190	.	.	.	0	0.00	0	0.00	0	0.00	400	0.67	C
292	Planctomycetes	Phycisphaerae	Phycisphaerales	.	.	81	0.13	0	0.00	311	0.49	0	0.00	C
1887	Planctomycetes	Phycisphaerae	Phycisphaerales	.	.	0	0.00	482	0.78	0	0.00	0	0.00	C
6655	Planctomycetes	Phycisphaerae	Phycisphaerales	.	.	0	0.00	200	0.32	0	0.00	0	0.00	C
10849	Planctomycetes	Phycisphaerae	Phycisphaerales	.	.	0	0.00	453	0.73	0	0.00	0	0.00	C
9420	Planctomycetes	Phycisphaerae	Pla1	.	.	89	0.14	74	0.12	0	0.00	0	0.00	C
659	Planctomycetes	Phycisphaerae	WD2101	.	.	500	0.78	0	0.00	678	1.07	0	0.00	C
2400	Planctomycetes	Phycisphaerae	WD2101	.	.	1833	2.86	0	0.00	172	0.27	0	0.00	C
10383	Planctomycetes	Phycisphaerae	WD2101	.	.	0	0.00	0	0.00	0	0.00	100	0.17	C
2964	Planctomycetes	Planctomycetia	Gemmatales	Gemmataceae	.	339	0.53	99	0.16	162	0.26	0	0.00	C
3075	Planctomycetes	Planctomycetia	Gemmatales	Gemmataceae	.	0	0.00	0	0.00	191	0.30	0	0.00	C
6290	Planctomycetes	Planctomycetia	Gemmatales	Gemmataceae	.	0	0.00	144	0.23	0	0.00	0	0.00	C
7458	Planctomycetes	Planctomycetia	Gemmatales	Gemmataceae	.	0	0.00	286	0.46	0	0.00	0	0.00	C
8237	Planctomycetes	Planctomycetia	Gemmatales	Gemmataceae	.	1281	2.00	0	0.00	0	0.00	0	0.00	E
9397	Planctomycetes	Planctomycetia	Gemmatales	Gemmataceae	.	0	0.00	212	0.34	0	0.00	0	0.00	C

1397	Planctomycetes	Planctomycetia	Gemmatales	Gemmataceae	Gemmata	0	0.00	0	0.00	0	0.00	113	0.19	C
2475	Planctomycetes	Planctomycetia	Gemmatales	Gemmataceae	Gemmata	0	0.00	0	0.00	151	0.24	0	0.00	C
10132	Planctomycetes	Planctomycetia	Gemmatales	Gemmataceae	Gemmata	0	0.00	76	0.12	0	0.00	0	0.00	C
10768	Planctomycetes	Planctomycetia	Gemmatales	Gemmataceae	Gemmata	137	0.21	0	0.00	729	1.15	0	0.00	C
10853	Planctomycetes	Planctomycetia	Gemmatales	Gemmataceae	Gemmata	0	0.00	74	0.12	0	0.00	0	0.00	C
5525	Planctomycetes	Planctomycetia	Gemmatales	Isosphaeraceae	.	81	0.13	0	0.00	0	0.00	0	0.00	E
7086	Planctomycetes	Planctomycetia	Gemmatales	Isosphaeraceae	.	86	0.13	0	0.00	0	0.00	0	0.00	E
3089	Planctomycetes	Planctomycetia	Pirellulales	Pirellulaceae	.	181	0.28	0	0.00	0	0.00	0	0.00	E
3405	Planctomycetes	Planctomycetia	Pirellulales	Pirellulaceae	.	0	0.00	0	0.00	0	0.00	128	0.21	C
4485	Planctomycetes	Planctomycetia	Pirellulales	Pirellulaceae	.	0	0.00	0	0.00	0	0.00	376	0.63	C
5877	Planctomycetes	Planctomycetia	Pirellulales	Pirellulaceae	.	0	0.00	0	0.00	0	0.00	226	0.38	C
7095	Planctomycetes	Planctomycetia	Pirellulales	Pirellulaceae	.	0	0.00	99	0.16	0	0.00	0	0.00	C
8859	Planctomycetes	Planctomycetia	Pirellulales	Pirellulaceae	.	98	0.15	0	0.00	0	0.00	0	0.00	E
7696	Planctomycetes	Planctomycetia	Pirellulales	Pirellulaceae	A17	0	0.00	324	0.52	0	0.00	110	0.18	C
3835	Planctomycetes	Planctomycetia	Pirellulales	Pirellulaceae	Pirellula	0	0.00	0	0.00	502	0.79	113	0.19	C
689	Planctomycetes	Planctomycetia	Planctomycetales	Planctomycetaceae	Planctomyces	0	0.00	0	0.00	0	0.00	125	0.21	C
1819	Planctomycetes	Planctomycetia	Planctomycetales	Planctomycetaceae	Planctomyces	0	0.00	238	0.39	0	0.00	0	0.00	C
2274	Planctomycetes	Planctomycetia	Planctomycetales	Planctomycetaceae	Planctomyces	114	0.18	1838	2.98	147	0.23	0	0.00	C
3061	Planctomycetes	Planctomycetia	Planctomycetales	Planctomycetaceae	Planctomyces	0	0.00	0	0.00	0	0.00	185	0.31	C
3668	Planctomycetes	Planctomycetia	Planctomycetales	Planctomycetaceae	Planctomyces	0	0.00	107	0.17	0	0.00	0	0.00	C
5215	Planctomycetes	Planctomycetia	Planctomycetales	Planctomycetaceae	Planctomyces	234	0.36	88	0.14	134	0.21	135	0.23	C
6808	Planctomycetes	Planctomycetia	Planctomycetales	Planctomycetaceae	Planctomyces	414	0.65	119	0.19	221	0.35	0	0.00	C
7702	Planctomycetes	Planctomycetia	Planctomycetales	Planctomycetaceae	Planctomyces	145	0.23	92	0.15	0	0.00	100	0.17	C
1400	Planctomycetes	vadinHA49	p04_C01	.	.	0	0.00	859	1.39	0	0.00	0	0.00	C
6657	Planctomycetes	vadinHA49	p04_C01	.	.	0	0.00	571	0.93	0	0.00	1713	2.87	C
1349	Proteobacteria	Alphaproteobacteria	.	.	.	117	0.18	0	0.00	316	0.50	0	0.00	C
6474	Proteobacteria	Alphaproteobacteria	.	.	.	0	0.00	99	0.16	0	0.00	0	0.00	C
766	Proteobacteria	Alphaproteobacteria	Caulobacterales	Caulobacteraceae	.	0	0.00	0	0.00	0	0.00	89	0.15	C
7581	Proteobacteria	Alphaproteobacteria	Caulobacterales	Caulobacteraceae	Phenylobacterium	103	0.16	0	0.00	0	0.00	0	0.00	E

1897	Proteobacteria	Alphaproteobacteria	Ellin329	.	.	470	0.73	86	0.14	425	0.67	185	0.31	C
29	Proteobacteria	Alphaproteobacteria	Rhizobiales	.	.	0	0.00	0	0.00	155	0.25	103	0.17	C
40	Proteobacteria	Alphaproteobacteria	Rhizobiales	.	.	0	0.00	80	0.13	301	0.48	76	0.13	C
3040	Proteobacteria	Alphaproteobacteria	Rhizobiales	.	.	114	0.18	0	0.00	235	0.37	109	0.18	C
3809	Proteobacteria	Alphaproteobacteria	Rhizobiales	.	.	0	0.00	343	0.56	0	0.00	0	0.00	C
6286	Proteobacteria	Alphaproteobacteria	Rhizobiales	.	.	156	0.24	120	0.19	139	0.22	114	0.19	C
6967	Proteobacteria	Alphaproteobacteria	Rhizobiales	.	.	0	0.00	0	0.00	102	0.16	0	0.00	C
7561	Proteobacteria	Alphaproteobacteria	Rhizobiales	.	.	182	0.28	0	0.00	82	0.13	0	0.00	C
7817	Proteobacteria	Alphaproteobacteria	Rhizobiales	.	.	0	0.00	0	0.00	0	0.00	95	0.16	C
6107	Proteobacteria	Alphaproteobacteria	Rhizobiales	Bradyrhizobiaceae	.	0	0.00	0	0.00	113	0.18	81	0.14	C
7088	Proteobacteria	Alphaproteobacteria	Rhizobiales	Hyphomicrobiaceae	Devosia	116	0.18	0	0.00	0	0.00	110	0.18	C
4390	Proteobacteria	Alphaproteobacteria	Rhizobiales	Hyphomicrobiaceae	Hyphomicrobium	108	0.17	158	0.26	143	0.23	0	0.00	B
10847	Proteobacteria	Alphaproteobacteria	Rhizobiales	Hyphomicrobiaceae	Rhodoplanes	0	0.00	0	0.00	0	0.00	75	0.13	C
5039	Proteobacteria	Alphaproteobacteria	Rhizobiales	Phyllobacteriaceae	Aminobacter	82	0.13	0	0.00	0	0.00	0	0.00	E
5665	Proteobacteria	Alphaproteobacteria	Rhizobiales	Phyllobacteriaceae	Mesorhizobium	0	0.00	77	0.12	0	0.00	283	0.47	C
1479	Proteobacteria	Alphaproteobacteria	Rhizobiales	Rhizobiaceae	.	0	0.00	0	0.00	0	0.00	77	0.13	A/B
4010	Proteobacteria	Alphaproteobacteria	Rhizobiales	Rhizobiaceae	.	0	0.00	0	0.00	0	0.00	83	0.14	A/B
4960	Proteobacteria	Alphaproteobacteria	Rhizobiales	Rhizobiaceae	.	0	0.00	0	0.00	465	0.74	218	0.36	A/B
8917	Proteobacteria	Alphaproteobacteria	Rhizobiales	Xanthobacteraceae	Labrys	0	0.00	0	0.00	0	0.00	158	0.26	C
217	Proteobacteria	Alphaproteobacteria	Rhodobacterales	Hyphomonadaceae	.	0	0.00	0	0.00	187	0.30	204	0.34	A/B
4447	Proteobacteria	Alphaproteobacteria	Rhodobacterales	Rhodobacteraceae	Amaricoccus	0	0.00	0	0.00	196	0.31	75	0.13	C
4468	Proteobacteria	Alphaproteobacteria	Rhodobacterales	Rhodobacteraceae	Paracoccus	94	0.15	0	0.00	550	0.87	0	0.00	C
6412	Proteobacteria	Alphaproteobacteria	Rhodobacterales	Rhodobacteraceae	Rhodobacter	80	0.12	0	0.00	147	0.23	119	0.20	B
8999	Proteobacteria	Alphaproteobacteria	Rhodobacterales	Rhodobacteraceae	Rhodobacter	0	0.00	0	0.00	110	0.17	0	0.00	A/B
1634	Proteobacteria	Alphaproteobacteria	Rhodospirillales	.	.	181	0.28	0	0.00	158	0.25	0	0.00	C
3085	Proteobacteria	Alphaproteobacteria	Rhodospirillales	Acetobacteraceae	.	105	0.16	0	0.00	0	0.00	0	0.00	E
9368	Proteobacteria	Alphaproteobacteria	Rhodospirillales	Acetobacteraceae	.	91	0.14	0	0.00	79	0.12	0	0.00	C
377	Proteobacteria	Alphaproteobacteria	Rhodospirillales	Rhodospirillaceae	.	0	0.00	458	0.74	0	0.00	0	0.00	C
4308	Proteobacteria	Alphaproteobacteria	Rhodospirillales	Rhodospirillaceae	.	0	0.00	0	0.00	0	0.00	75	0.13	C

4868	Proteobacteria	Alphaproteobacteria	Rhodospirillales	Rhodospirillaceae	.	106	0.17	0	0.00	188	0.30	0	0.00	C
6473	Proteobacteria	Alphaproteobacteria	Rhodospirillales	Rhodospirillaceae	.	0	0.00	0	0.00	0	0.00	159	0.27	C
7608	Proteobacteria	Alphaproteobacteria	Rhodospirillales	Rhodospirillaceae	.	0	0.00	398	0.64	0	0.00	120	0.20	C
8434	Proteobacteria	Alphaproteobacteria	Rhodospirillales	Rhodospirillaceae	.	0	0.00	166	0.27	0	0.00	0	0.00	C
8726	Proteobacteria	Alphaproteobacteria	Rhodospirillales	Rhodospirillaceae	.	0	0.00	153	0.25	0	0.00	0	0.00	C
8756	Proteobacteria	Alphaproteobacteria	Rhodospirillales	Rhodospirillaceae	.	187	0.29	98	0.16	0	0.00	200	0.33	C
10700	Proteobacteria	Alphaproteobacteria	Rhodospirillales	Rhodospirillaceae	.	187	0.29	0	0.00	0	0.00	85	0.14	C
4488	Proteobacteria	Alphaproteobacteria	Rickettsiales	.	.	0	0.00	0	0.00	0	0.00	446	0.75	C
6676	Proteobacteria	Alphaproteobacteria	Rickettsiales	.	.	118	0.18	0	0.00	0	0.00	0	0.00	E
6525	Proteobacteria	Alphaproteobacteria	Sphingomonadales	Sphingomonadaceae	.	129	0.20	0	0.00	239	0.38	100	0.17	B
6610	Proteobacteria	Alphaproteobacteria	Sphingomonadales	Sphingomonadaceae	.	0	0.00	148	0.24	84	0.13	777	1.30	A/B
7854	Proteobacteria	Alphaproteobacteria	Sphingomonadales	Sphingomonadaceae	.	91	0.14	0	0.00	0	0.00	0	0.00	D
7855	Proteobacteria	Alphaproteobacteria	Sphingomonadales	Sphingomonadaceae	.	156	0.24	0	0.00	155	0.25	166	0.28	B
9262	Proteobacteria	Alphaproteobacteria	Sphingomonadales	Sphingomonadaceae	Sphingomonas	0	0.00	0	0.00	125	0.20	471	0.79	A/B
4793	Proteobacteria	Alphaproteobacteria	Sphingomonadales	Sphingomonadaceae	Sphingomonas	0	0.00	0	0.00	0	0.00	261	0.44	A/B
8133	Proteobacteria	Betaproteobacteria	.	.	.	0	0.00	0	0.00	0	0.00	200	0.33	C
5076	Proteobacteria	Betaproteobacteria	Burkholderiales	.	.	0	0.00	0	0.00	114	0.18	0	0.00	C
7118	Proteobacteria	Betaproteobacteria	Burkholderiales	.	.	0	0.00	202	0.33	0	0.00	223	0.37	C
1182	Proteobacteria	Betaproteobacteria	Burkholderiales	Burkholderiaceae	Pandoraea	0	0.00	0	0.00	130	0.21	0	0.00	C
462	Proteobacteria	Betaproteobacteria	Burkholderiales	Comamonadaceae	.	80	0.12	0	0.00	0	0.00	0	0.00	D
703	Proteobacteria	Betaproteobacteria	Burkholderiales	Comamonadaceae	.	0	0.00	0	0.00	181	0.29	362	0.61	A/B
1346	Proteobacteria	Betaproteobacteria	Burkholderiales	Comamonadaceae	.	1847	2.88	472	0.76	2486	3.93	0	0.00	B
1486	Proteobacteria	Betaproteobacteria	Burkholderiales	Comamonadaceae	.	0	0.00	0	0.00	97	0.15	0	0.00	A/B
1894	Proteobacteria	Betaproteobacteria	Burkholderiales	Comamonadaceae	.	170	0.26	0	0.00	234	0.37	0	0.00	B
2144	Proteobacteria	Betaproteobacteria	Burkholderiales	Comamonadaceae	.	0	0.00	0	0.00	78	0.12	0	0.00	A/B
2604	Proteobacteria	Betaproteobacteria	Burkholderiales	Comamonadaceae	.	309	0.48	1520	2.46	100	0.16	979	1.64	B
3119	Proteobacteria	Betaproteobacteria	Burkholderiales	Comamonadaceae	.	84	0.13	0	0.00	0	0.00	0	0.00	D
3295	Proteobacteria	Betaproteobacteria	Burkholderiales	Comamonadaceae	.	115	0.18	193	0.31	0	0.00	96	0.16	B
4931	Proteobacteria	Betaproteobacteria	Burkholderiales	Comamonadaceae	.	0	0.00	97	0.16	0	0.00	0	0.00	A/

														B
5267	Proteobacteria	Betaproteobacteria	Burkholderiales	Comamonadaceae	.	547	0.85	750	1.22	520	0.82	602	1.01	B
8695	Proteobacteria	Betaproteobacteria	Burkholderiales	Comamonadaceae	.	91	0.14	0	0.00	327	0.52	326	0.55	B
10333	Proteobacteria	Betaproteobacteria	Burkholderiales	Comamonadaceae	.	0	0.00	73	0.12	0	0.00	115	0.19	A/B
11198	Proteobacteria	Betaproteobacteria	Burkholderiales	Comamonadaceae	.	0	0.00	0	0.00	0	0.00	77	0.13	A/B
7850	Proteobacteria	Betaproteobacteria	Burkholderiales	Comamonadaceae	Azohydromonas	0	0.00	0	0.00	114	0.18	458	0.77	C
1471	Proteobacteria	Betaproteobacteria	Burkholderiales	Comamonadaceae	Diaphorobacter	1671	2.60	0	0.00	958	1.52	648	1.08	C
4578	Proteobacteria	Betaproteobacteria	Burkholderiales	Comamonadaceae	Hydrogenophaga	220	0.34	0	0.00	831	1.31	0	0.00	C
4082	Proteobacteria	Betaproteobacteria	Burkholderiales	Comamonadaceae	Methylibium	0	0.00	166	0.27	159	0.25	130	0.22	C
376	Proteobacteria	Betaproteobacteria	Burkholderiales	Oxalobacteraceae	.	0	0.00	791	1.28	0	0.00	800	1.34	C
813	Proteobacteria	Betaproteobacteria	Burkholderiales	Oxalobacteraceae	.	0	0.00	278	0.45	0	0.00	188	0.31	C
1886	Proteobacteria	Betaproteobacteria	Burkholderiales	Oxalobacteraceae	.	146	0.23	178	0.29	0	0.00	202	0.34	C
8640	Proteobacteria	Betaproteobacteria	Burkholderiales	Oxalobacteraceae	.	0	0.00	0	0.00	0	0.00	265	0.44	C
3002	Proteobacteria	Betaproteobacteria	Burkholderiales	Oxalobacteraceae	Cupriavidus	0	0.00	0	0.00	0	0.00	333	0.56	C
7062	Proteobacteria	Betaproteobacteria	Burkholderiales	Oxalobacteraceae	Cupriavidus	0	0.00	0	0.00	0	0.00	1341	2.24	C
2149	Proteobacteria	Betaproteobacteria	IS-44	.	.	0	0.00	161	0.26	0	0.00	0	0.00	C
6788	Proteobacteria	Betaproteobacteria	IS-44	.	.	0	0.00	664	1.08	0	0.00	0	0.00	C
4389	Proteobacteria	Betaproteobacteria	Methylophilales	Methylophilaceae	.	167	0.26	0	0.00	158	0.25	0	0.00	C
6010	Proteobacteria	Betaproteobacteria	Methylophilales	Methylophilaceae	.	150	0.23	0	0.00	0	0.00	0	0.00	E
6313	Proteobacteria	Betaproteobacteria	MND1	.	.	0	0.00	84	0.14	0	0.00	138	0.23	C
2030	Proteobacteria	Betaproteobacteria	Nitrosomonadales	Nitrosomonadaceae	.	148	0.23	0	0.00	0	0.00	0	0.00	E
2609	Proteobacteria	Betaproteobacteria	Nitrosomonadales	Nitrosomonadaceae	.	0	0.00	334	0.54	0	0.00	0	0.00	C
6342	Proteobacteria	Betaproteobacteria	Nitrosomonadales	Nitrosomonadaceae	.	849	1.32	0	0.00	1469	2.32	0	0.00	C
5869	Proteobacteria	Betaproteobacteria	Rhodocyclales	Rhodocyclaceae	.	99	0.15	0	0.00	103	0.16	0	0.00	C
6465	Proteobacteria	Betaproteobacteria	Rhodocyclales	Rhodocyclaceae	.	0	0.00	79	0.13	0	0.00	273	0.46	C
7715	Proteobacteria	Betaproteobacteria	Rhodocyclales	Rhodocyclaceae	.	81	0.13	0	0.00	0	0.00	0	0.00	E
9029	Proteobacteria	Betaproteobacteria	Rhodocyclales	Rhodocyclaceae	.	0	0.00	0	0.00	181	0.29	0	0.00	C
9585	Proteobacteria	Betaproteobacteria	Rhodocyclales	Rhodocyclaceae	.	351	0.55	0	0.00	1101	1.74	0	0.00	C
1898	Proteobacteria	Betaproteobacteria	Rhodocyclales	Rhodocyclaceae	Zoogloea	283	0.44	0	0.00	0	0.00	0	0.00	E

2027	Proteobacteria	Betaproteobacteria	Rhodocyclales	Rhodocyclaceae	Zoogloea	0	0.00	0	0.00	1564	2.47	0	0.00	C
3642	Proteobacteria	Betaproteobacteria	SC-I-84	.	.	430	0.67	76	0.12	103	0.16	0	0.00	C
54	Proteobacteria	Deltaproteobacteria	Bdellovibrionales	Bdellovibrionaceae	Bdellovibrio	0	0.00	269	0.44	0	0.00	0	0.00	C
8687	Proteobacteria	Deltaproteobacteria	Bdellovibrionales	Bdellovibrionaceae	Bdellovibrio	201	0.31	0	0.00	0	0.00	0	0.00	E
10144	Proteobacteria	Deltaproteobacteria	Desulfuromonadales	Geobacteraceae	Geobacter	0	0.00	0	0.00	0	0.00	91	0.15	C
807	Proteobacteria	Deltaproteobacteria	Myxococcales	.	.	83	0.13	0	0.00	0	0.00	0	0.00	E
1340	Proteobacteria	Deltaproteobacteria	Myxococcales	.	.	0	0.00	0	0.00	0	0.00	116	0.19	C
1426	Proteobacteria	Deltaproteobacteria	Myxococcales	.	.	0	0.00	0	0.00	0	0.00	120	0.20	C
2399	Proteobacteria	Deltaproteobacteria	Myxococcales	.	.	175	0.27	0	0.00	0	0.00	0	0.00	E
2474	Proteobacteria	Deltaproteobacteria	Myxococcales	.	.	0	0.00	0	0.00	0	0.00	193	0.32	C
2894	Proteobacteria	Deltaproteobacteria	Myxococcales	.	.	0	0.00	0	0.00	0	0.00	98	0.16	C
5528	Proteobacteria	Deltaproteobacteria	Myxococcales	.	.	0	0.00	820	1.33	0	0.00	463	0.77	C
6434	Proteobacteria	Deltaproteobacteria	Myxococcales	.	.	148	0.23	0	0.00	0	0.00	0	0.00	E
6500	Proteobacteria	Deltaproteobacteria	Myxococcales	.	.	112	0.17	0	0.00	91	0.14	0	0.00	C
6508	Proteobacteria	Deltaproteobacteria	Myxococcales	.	.	0	0.00	271	0.44	0	0.00	0	0.00	C
6744	Proteobacteria	Deltaproteobacteria	Myxococcales	.	.	0	0.00	0	0.00	0	0.00	162	0.27	C
7824	Proteobacteria	Deltaproteobacteria	Myxococcales	.	.	0	0.00	238	0.39	0	0.00	103	0.17	C
8260	Proteobacteria	Deltaproteobacteria	Myxococcales	.	.	0	0.00	0	0.00	225	0.36	0	0.00	C
10736	Proteobacteria	Deltaproteobacteria	Myxococcales	.	.	0	0.00	0	0.00	108	0.17	0	0.00	C
8560	Proteobacteria	Deltaproteobacteria	Myxococcales	Myxococcaceae	Anaeromyxobacter	0	0.00	367	0.59	0	0.00	0	0.00	C
8674	Proteobacteria	Deltaproteobacteria	Syntrophobacterales	Syntrophobacteraceae	.	0	0.00	213	0.35	0	0.00	0	0.00	C
2777	Proteobacteria	Gammaproteobacteria	Aeromonadales	Aeromonadaceae	.	1044	1.63	0	0.00	3051	4.83	0	0.00	C
10094	Proteobacteria	Gammaproteobacteria	Alteromonadales	Alteromonadaceae	HB2-32-21	0	0.00	0	0.00	0	0.00	373	0.62	C
6594	Proteobacteria	Gammaproteobacteria	Alteromonadales	Alteromonadaceae	Marinobacter	0	0.00	0	0.00	121	0.19	0	0.00	C
696	Proteobacteria	Gammaproteobacteria	Chromatiales	.	.	0	0.00	0	0.00	0	0.00	82	0.14	C
5037	Proteobacteria	Gammaproteobacteria	Enterobacteriales	Enterobacteriaceae	Enterobacter	109	0.17	0	0.00	0	0.00	0	0.00	D
5793	Proteobacteria	Gammaproteobacteria	Legionellales	.	.	0	0.00	0	0.00	0	0.00	217	0.36	C
5986	Proteobacteria	Gammaproteobacteria	Legionellales	.	.	0	0.00	0	0.00	0	0.00	82	0.14	C

1469	Proteobacteria	Gammaproteobacteria	Legionellales	Coxiellaceae	.	126	0.20	0	0.00	0	0.00	0	0.00	E
3464	Proteobacteria	Gammaproteobacteria	Legionellales	Coxiellaceae	.	133	0.21	0	0.00	0	0.00	0	0.00	E
11061	Proteobacteria	Gammaproteobacteria	Legionellales	Coxiellaceae	.	0	0.00	117	0.19	0	0.00	0	0.00	C
11284	Proteobacteria	Gammaproteobacteria	Legionellales	Legionellaceae	Legionella	0	0.00	0	0.00	0	0.00	85	0.14	C
2875	Proteobacteria	Gammaproteobacteria	Pseudomonadales	Pseudomonadaceae	Pseudomonas	1091	1.70	4419	7.16	109	0.17	1200	2.01	B
3120	Proteobacteria	Gammaproteobacteria	Pseudomonadales	Pseudomonadaceae	Pseudomonas	118	0.18	319	0.52	0	0.00	0	0.00	B
2394	Proteobacteria	Gammaproteobacteria	Xanthomonadales	Sinobacteraceae	.	0	0.00	0	0.00	118	0.19	0	0.00	C
2493	Proteobacteria	Gammaproteobacteria	Xanthomonadales	Sinobacteraceae	.	92	0.14	0	0.00	0	0.00	0	0.00	E
3287	Proteobacteria	Gammaproteobacteria	Xanthomonadales	Sinobacteraceae	.	91	0.14	0	0.00	0	0.00	0	0.00	E
6317	Proteobacteria	Gammaproteobacteria	Xanthomonadales	Sinobacteraceae	.	152	0.24	415	0.67	224	0.35	139	0.23	C
8473	Proteobacteria	Gammaproteobacteria	Xanthomonadales	Sinobacteraceae	.	113	0.18	0	0.00	0	0.00	0	0.00	E
9694	Proteobacteria	Gammaproteobacteria	Xanthomonadales	Sinobacteraceae	.	0	0.00	468	0.76	0	0.00	238	0.40	C
4600	Proteobacteria	Gammaproteobacteria	Xanthomonadales	Sinobacteraceae	Steroidobacter	142	0.22	0	0.00	206	0.33	0	0.00	C
8223	Proteobacteria	Gammaproteobacteria	Xanthomonadales	Xanthomonadaceae	.	205	0.32	884	1.43	596	0.94	824	1.38	C
8241	Proteobacteria	Gammaproteobacteria	Xanthomonadales	Xanthomonadaceae	.	604	0.94	113	0.18	813	1.29	114	0.19	C
9544	Proteobacteria	Gammaproteobacteria	Xanthomonadales	Xanthomonadaceae	.	0	0.00	104	0.17	0	0.00	113	0.19	C
3886	Proteobacteria	Gammaproteobacteria	Xanthomonadales	Xanthomonadaceae	Dokdonella	545	0.85	0	0.00	244	0.39	0	0.00	C
7701	Proteobacteria	Gammaproteobacteria	Xanthomonadales	Xanthomonadaceae	Dokdonella	0	0.00	0	0.00	0	0.00	643	1.08	C
5967	Proteobacteria	Gammaproteobacteria	Xanthomonadales	Xanthomonadaceae	Luteibacter	0	0.00	0	0.00	0	0.00	148	0.25	C
10564	Proteobacteria	Gammaproteobacteria	Xanthomonadales	Xanthomonadaceae	Lysobacter	0	0.00	0	0.00	110	0.17	0	0.00	C
4386	Proteobacteria	Gammaproteobacteria	Xanthomonadales	Xanthomonadaceae	Lysobacter	247	0.38	0	0.00	182	0.29	3141	5.26	C
1967	Proteobacteria	Gammaproteobacteria	Xanthomonadales	Xanthomonadaceae	Pseudoxanthomonas	0	0.00	0	0.00	0	0.00	98	0.16	C
2246	Proteobacteria	Gammaproteobacteria	Xanthomonadales	Xanthomonadaceae	Thermomonas	127	0.20	0	0.00	456	0.72	79	0.13	C
103	Spirochaetes	[Leptospirae]	[Leptospirales]	Leptospiraceae	Turneriella	0	0.00	0	0.00	0	0.00	88	0.15	C
3795	TM7	TM7-1	.	.	.	168	0.26	0	0.00	0	0.00	0	0.00	E
4289	TM7	TM7-1	.	.	.	155	0.24	0	0.00	0	0.00	0	0.00	E
6674	TM7	TM7-1	.	.	.	84	0.13	98	0.16	0	0.00	0	0.00	C

10434	TM7	TM7-1	.	.	.	0	0.00	0	0.00	0	0.00	157	0.26	C
10562	TM7	TM7-3	.	.	.	0	0.00	0	0.00	0	0.00	126	0.21	C
4879	TM7	TM7-3	EW055	.	.	0	0.00	0	0.00	0	0.00	118	0.20	C
5211	Verrucomicrobia	[Pedosphaerae]	[Pedosphaerales]	.	.	216	0.34	0	0.00	0	0.00	0	0.00	E
6835	Verrucomicrobia	[Pedosphaerae]	[Pedosphaerales]	.	.	0	0.00	0	0.00	0	0.00	123	0.21	C
7479	Verrucomicrobia	[Pedosphaerae]	[Pedosphaerales]	.	.	286	0.45	0	0.00	0	0.00	104	0.17	C
9829	Verrucomicrobia	[Pedosphaerae]	[Pedosphaerales]	.	.	138	0.22	0	0.00	0	0.00	0	0.00	E
11135	Verrucomicrobia	[Pedosphaerae]	[Pedosphaerales]	.	.	724	1.13	0	0.00	0	0.00	0	0.00	E
5596	Verrucomicrobia	[Pedosphaerae]	[Pedosphaerales]	auto67_4W	.	0	0.00	181	0.29	0	0.00	253	0.42	C
7468	Verrucomicrobia	[Pedosphaerae]	[Pedosphaerales]	auto67_4W	.	425	0.66	0	0.00	849	1.34	326	0.55	C
9080	Verrucomicrobia	[Pedosphaerae]	[Pedosphaerales]	auto67_4W	.	611	0.95	0	0.00	0	0.00	84	0.14	C
1855	Verrucomicrobia	[Pedosphaerae]	[Pedosphaerales]	Ellin515	.	0	0.00	78	0.13	0	0.00	0	0.00	C
5237	Verrucomicrobia	[Pedosphaerae]	[Pedosphaerales]	Ellin515	.	91	0.14	0	0.00	0	0.00	0	0.00	E
5498	Verrucomicrobia	[Pedosphaerae]	[Pedosphaerales]	Ellin515	.	148	0.23	0	0.00	0	0.00	0	0.00	E
7920	Verrucomicrobia	[Pedosphaerae]	[Pedosphaerales]	Ellin517	.	0	0.00	0	0.00	0	0.00	247	0.41	C
8886	Verrucomicrobia	[Pedosphaerae]	[Pedosphaerales]	Ellin517	.	0	0.00	0	0.00	0	0.00	254	0.43	C
3785	Verrucomicrobia	[Pedosphaerae]	[Pedosphaerales]	R4-41B	.	313	0.49	177	0.29	146	0.23	0	0.00	C
10877	Verrucomicrobia	[Pedosphaerae]	[Pedosphaerales]	R4-41B	.	0	0.00	0	0.00	0	0.00	220	0.37	C
1717	Verrucomicrobia	[Spartobacteria]	[Chthoniobacterales]	[Chthoniobacteraceae]	.	147	0.23	0	0.00	0	0.00	0	0.00	E
2128	Verrucomicrobia	[Spartobacteria]	[Chthoniobacterales]	[Chthoniobacteraceae]	.	179	0.28	0	0.00	0	0.00	0	0.00	E
8885	Verrucomicrobia	[Spartobacteria]	[Chthoniobacterales]	[Chthoniobacteraceae]	.	0	0.00	133	0.22	0	0.00	0	0.00	C
10108	Verrucomicrobia	[Spartobacteria]	[Chthoniobacterales]	[Chthoniobacteraceae]	Chthoniobacter	80	0.12	129	0.21	0	0.00	275	0.46	C
10641	Verrucomicrobia	[Spartobacteria]	[Chthoniobacterales]	[Chthoniobacteraceae]	Chthoniobacter	0	0.00	0	0.00	0	0.00	127	0.21	C
1842	Verrucomicrobia	Opitutae	Opitutales	Opitutaceae	.	238	0.37	0	0.00	0	0.00	0	0.00	E
3098	Verrucomicrobia	Opitutae	Opitutales	Opitutaceae	Opitutus	0	0.00	174	0.28	0	0.00	174	0.29	C
3144	Verrucomicrobia	Opitutae	Opitutales	Opitutaceae	Opitutus	636	0.99	0	0.00	102	0.16	190	0.32	C
3172	Verrucomicrobia	Opitutae	Opitutales	Opitutaceae	Opitutus	188	0.29	219	0.35	408	0.65	172	0.29	C

7288	Verrucomicrobia	Verrucomicrobiae	Verrucomicrobiales	Verrucomicrobiaceae	.	97	0.15	0	0.00	0	0.00	0	0.00	E
8576	Verrucomicrobia	Verrucomicrobiae	Verrucomicrobiales	Verrucomicrobiaceae	.	0	0.00	0	0.00	0	0.00	159	0.27	C
958	Verrucomicrobia	Verrucomicrobiae	Verrucomicrobiales	Verrucomicrobiaceae	Luteolibacter	1167	1.82	0	0.00	1445	2.29	0	0.00	C
5503	Verrucomicrobia	Verrucomicrobiae	Verrucomicrobiales	Verrucomicrobiaceae	Luteolibacter	213	0.33	0	0.00	0	0.00	0	0.00	E
8862	Verrucomicrobia	Verrucomicrobiae	Verrucomicrobiales	Verrucomicrobiaceae	Prosthecobacter	0	0.00	507	0.82	0	0.00	0	0.00	C
9684	Verrucomicrobia	Verrucomicrobiae	Verrucomicrobiales	Verrucomicrobiaceae	Prosthecobacter	0	0.00	0	0.00	625	0.99	0	0.00	C
6269	WPS-2	.	.	.	.	0	0.00	74	0.12	0	0.00	0	0.00	C
1000	WS3	PRR-12	Sediment-1	PRR-10	.	103	0.16	0	0.00	0	0.00	0	0.00	E

Table S4 Conservation of putative genes related to Mn(II) oxidation

Genes (accession)	*Comamonas testosteroni*				*Delftia acidovorans*		*Acidovorax* sp.		*Leptothrix discophora*	*Pseudomonas putida*
	KF-1	ATCC 11996	S44	CNB-1	SPH-1	NBRC 14950	CF316	Root 219	SS-1	GB-1
moxA (CAJ19378.1)	+ [a]	+	-	-	+	+	+	+	Unknown[b]	+
mofA (CAA81037.2)	-	-	-	-	-	-	-	-	+	+
mopA (EAS51309.1)	-	-	-	-	-	-	-	-	Unknown	+
mnxG (AAB06489.1)	-	-	-	-	-	-	-	-	Unknown	+

[a] The homology of e-values ≤ E^{-15} was estimated '+' using BLASTP analysis

[b] Whole genome information for *Leptothrix discophora* SS-1 has not yet been registered in Genbank, even at draft level
